# Evaluation of steroids for acute COVID in the prevention of long COVID in children: An EHR and pediatric cohort study from the RECOVER Initiative

**DOI:** 10.1371/journal.pone.0350888

**Published:** 2026-06-18

**Authors:** Kathryn Hirabayashi, Vitaly Lorman, Shannon Wuller, Leyna V. Aragon, Ravi Jhaveri, Nita Jain, John Erik Leikauf, Jennifer A. Muszynski, Aaron Thomas Martinez, Miranda Higginbotham, Payal B. Patel, Marc A. Sala, Grant S. Schulert, Mei Liu, Stacey Knight, Elizabeth A. Chrischilles, Yuriy Bisyuk, Bradley W. Taylor, Abu Saleh Mohammad Mosa, Sandy L. Gonzalez, Matthew Authement, Wyatt P. Bensken, Daniel Fort, Soledad A. Fernandez, Jonathan Arnold, Michael J. Becich, Wenke Hwang, Mollie R. Cummins, Susan Kim, Yacob G. Tedla, L. Charles Bailey, Christopher B. Forrest, Suchitra Rao, on behalf of the RECOVER Consortium

**Affiliations:** 1 Applied Clinical Research Center, Children’s Hospital of Philadelphia, Philadelphia, Pennsylvania, United States of America; 2 Department of Population Health, New York University Grossman School of Medicine, New York, New York, United States of America; 3 University of New Mexico Health Sciences Center, Albuquerque, New Mexico, United States of America; 4 RECOVER Patient, Caregiver, or Community Advocate Representative, New York, New York, United States of America; 5 Division of Infectious Diseases, Ann and Robert H. Lurie Children’s Hospital of Chicago, Chicago, Illinois, United States of America; 6 Timeless Biosciences, Atlanta, Georgia, United States of America; 7 Department of Psychiatry and Behavioral Sciences, Division of Child and Adolescent Psychiatry and Child Development, Stanford University School of Medicine, Palo Alto, California, United States of America; 8 Department of Pediatrics, Division of Critical Care Medicine, Nationwide Children’s Hospital, Columbus, Ohio, United States of America; 9 Department of Neurology, Seattle Children's Hospital, Seattle, Washington, United States of America; 10 Division of Pulmonary and Critical Care Medicine, Northwestern University Feinberg School of Medicine, Chicago, Illinois, United States of America; 11 Department of Pediatrics, Division of Rheumatology, Cincinnati Children’s Hospital Medical Center, Cincinnati, Ohio, United States of America; 12 Department of Health Outcomes and Biomedical Informatics, University of Florida, College of Medicine, GainesvilleFlorida, United States of America; 13 Intermountain Medical Center Heart Institute, Intermountain Health, Salt Lake City, Utah, United States of America; 14 Department of Epidemiology, College of Public Health, The University of Iowa, Iowa City, Iowa, United States of America; 15 University Medical Center New Orleans, New Orleans, Los Angeles, United States of America; 16 Clinical and Translational Science Institute, The Medical College of Wisconsin, Milwaukee, Wisconsin, United States of America; 17 Department of Biomedical Informatics and Data Science, Heersink School of Medicine, The University of Alabama at Birmingham, Alabama; 18 Nicklaus Children’s Hospital, Miami, Florida, United States of America; 19 Division of Pediatrics, University of Nebraska Medical Center, Omaha, Nebraska, United States of America; 20 OCHIN, Inc., Portland, Oregon, United States of America; 21 Center for Outcomes Research, Ochsner Health, New Orleans, Louisiana, United States of America; 22 Department of Biomedical Informatics and Center for Biostatistics, Ohio State University, Columbus, Ohio, United States of America; 23 Division of General Internal Medicine, University of Pittsburgh School of Medicine, Pittsburgh, Pennsylvania, United States of America; 24 Department of Biomedical Informatics, University of Pittsburgh School of Medicine., Pittsburgh, Pennsylvania, United States of America; 25 Department of Public Health Sciences, Penn State University College of Medicine, Hershey, Pennsylvania, United States of America; 26 College of Nursing, University of Utah, Salt Lake City, Utah, United States of America; 27 Division of Rheumatology, University of California San Francisco; Benioff Children’s Hospital, San Francisco, California, United States of America; 28 Department of Medicine, Division of Epidemiology, Vanderbilt University Medical Center, Nashville, Tennessee, United States of America; 29 Department of Pediatrics, University of Colorado School of Medicine and Children’s Hospital, Aurora, Colorado, United States of America; University of Colombo Faculty of Medicine, SRI LANKA

## Abstract

**Background:**

Studies have shown that use of immunomodulators during the acute phase of SARS-CoV-2 infection may decrease development of post-acute sequelae of SARS-CoV-2 (PASC) or long COVID; however, such studies have not been conducted in children.

**Objective:**

Evaluate the effectiveness of steroid use during the acute phase of SARS-CoV-2 infection in preventing long COVID in children.

**Methods:**

We conducted a retrospective cohort study using target trial emulation methodology to compare children and youth who did and did not receive dexamethasone, prednisone, prednisolone or methylprednisolone within 12 days of SARS-CoV-2 infection. Inverse propensity of treatment weighting was used to balance covariates between treated and untreated patients in hospitalized and outpatient groups. The primary outcome was the development of PASC in the 1–6 months following acute infection using a computable phenotype definition. Secondary outcomes included respiratory, musculoskeletal, gastrointestinal and neurological subphenotypes and the PASC ICD-10-CM diagnosis code. We calculated hazard ratios from Cox proportional models with 95% confidence intervals.

**Results:**

From a starting cohort of 854,128 children/youth, of whom 768,845 (90.0%) were outpatients and 85,283 (10.0%) were inpatients at the time of SARS-CoV-2 infection, the weighted outpatient cohort included 22,085 steroid-treated children and 20,373 in the non-steroid group. Following weighting, the hospitalized cohort included 11,250 steroid-treated children and 10,340 untreated children. In hospitalized patients, there were no significant treatment differences in the development of PASC in the 1−6 months following acute SARS-CoV-2 infection except for a lower risk of gastrointestinal PASC in treated patients (HR: 0.58; [95% CI: 0.39–0.85], p = 0.01). In outpatients, no treatment differences were observed in the development of PASC subphenotypes.

**Conclusions:**

Steroids administered during acute SARS-CoV-2 infection did not lead to a decreased risk of PASC, with the exception of gastrointestinal presentations. Additional studies are needed to confirm the benefit of steroids and other immunomodulators in preventing long COVID.

## Introduction

The SARS-CoV-2 virus continues to cause significant morbidity and mortality worldwide. There is a growing cohort of individuals suffering with persistent symptoms after acute infection, termed post-acute sequelae of SARS-CoV-2 (PASC) or long COVID. The US National Academies of Sciences, Engineering, and Medicine defines long COVID as “an infection-associated chronic condition that occurs after severe acute respiratory syndrome Coronavirus 2 (SARS-CoV-2) infection, present for at least three months, manifesting as continuous, relapsing, or progressive disease affecting one or more organ systems” [1]. The clinical manifestations of long COVID are heterogeneous, and have been well-recognized in children as well as adults, with up to 10% of children experiencing a symptom of long COVID at least 1 month after acute infection. Trials exploring treatments for PASC are urgently needed but are lacking in children.

While multiple underlying mechanisms have been proposed, including viral persistence and endothelial dysfunction, chronic inflammation secondary to the initial aberrant immune response has been thought to be a predominant mechanism. Drugs with anti-inflammatory properties such as corticosteroids have been suggested as a potential treatment to prevent symptoms of long COVID. Corticosteroids are indicated for use for severe COVID, with evidence of reducing mortality among patients with severe COVID [2]. Emerging evidence demonstrates the benefit of steroids in reducing persistent long COVID symptoms in adults [3–5]. However, there are no studies evaluating the impact of steroids against long COVID in children to date. In the absence of clinical trial data, target trial emulation uses statistical analysis of existing EHR data to mimic a randomized controlled trial and evaluate potential treatment benefits. In this retrospective cohort study, using a target trial emulation methodology, we propose to evaluate the effectiveness of steroids used for acute COVID-19 infection in the prevention of long COVID in children. We hypothesize that steroids administered at the time of acute COVID infection have a protective effect against the development of long COVID in children.

## Methods

### Data source

We conducted this retrospective target trial emulation as part of the NIH Researching COVID to Enhance Recovery (RECOVER) Initiative, which seeks to understand, treat, and prevent the post-acute sequelae of SARS-CoV-2 infection (PASC) (S1 Table) [6]. We used electronic health record (EHR) data from 35 pediatric sites contributing to the Patient-Centered Clinical Research Networks (PCORnet^®^) [7], which is a US-based network of health systems with EHR data standardized within eight large clinical research networks [8]. Institutional Review Board (IRB) approval was obtained under Biomedical Research Alliance of New York (BRANY) protocol #21–08–508. BRANY waived the need for consent and HIPAA authorization. The data was accessed for research purposes on August 9, 2025. Authors had access to individual-level data; however, individual participants were de-identified.

We used EHR data from all healthcare encounters in outpatient, inpatient, and emergency department settings from the included health systems. Data were extracted from the RECOVER/PCORnet Database version s12 and included EHR data with dates of service through September 2024. Reporting of study design and results follows the reporting of studies conducted using the transparent reporting of observational studies emulating a Target Trial (TARGET) guideline [9].

### Cohorts

Our cohort comprised children/youth under 19 years of age with a positive SARS-CoV-2 viral test (PCR or antigen), or who had a COVID-19/SARS-CoV-2 associated diagnosis or complication code, or received nirmatrelvir/ritonavir or remdesivir (case group) from March 1, 2020 to April 4, 2024 at sites with complete data, defined as the presence of usable COVID-19 testing, condition, encounter, and observation records. We excluded two sites with insufficient data capture of SARS CoV-2 testing or COVID-19 condition codes, or with notable missingness of observation or encounter data. We also excluded patients with multi-system inflammatory syndrome in children (MIS-C) and patients who were missing sex or date of birth information. For the primary analyses, we excluded those with prior steroid exposure (defined as any steroid exposure occurring within 1–90 days prior to the COVID date). Additional exclusions were applied to ensure that all patients were eligible for an outcome starting 28 days after the COVID date. Target trial emulation specifications as well as inclusion and exclusion criteria are outlined in further detail in **S2 and S3 Tables** in S2 File Time zero (T0) was identified as the date of treatment initiation in patients who received steroids. The distribution of the difference in days between COVID infection date and T0 was identified in treated patients, and we sampled from this distribution to generate corresponding T0 dates in the untreated group.

### Exposure

The main exposure of interest was steroid receipt during acute COVID, including intravenous or oral dexamethasone, methylprednisolone, prednisolone or prednisone (case group). We identified patients with treatment duration of 1–10 days, with receipt of these medications starting within 0–12 days following the COVID date. We considered this exposure window sufficiently narrow to reduce the likelihood of immortal time bias. The COVID date was defined as a random date of a positive SARS-CoV-2 test, diagnosis code or antiviral for treated and untreated patients. The control group comprised individuals without evidence of receipt of these medications in the 0–12 days following SARS-CoV-2 infection (as further outlined in **S2 and S3 Tables** in S2 File).

### Outcomes

The primary outcome of interest was long COVID in the 28–179 days (or 1–6 months) following acute infection. Long COVID was defined using our pediatric-specific computable phenotype for PASC [10], which was developed using a number of symptom and condition codes that have been reported to occur in children with long COVID based on our prior work (code set available at https://github.com/RECOVER-Coordinating-Center/steroids_long_covid). Secondary exploratory outcomes included specific sub-phenotypes of long COVID, including respiratory, musculoskeletal, gastrointestinal, neurologic/constitutional and the ICD-10 code for long COVID (U09.9), which were not mutually exclusive groups.

### Covariates and stratification

Patients were stratified by hospitalization status at the time of their SARS-CoV-2 cohort entry date (hospitalized; outpatient, including ED visits) and analyses were performed within each stratum. Covariates included site, age group at T0, sex, race, ethnicity, 3-month T0 period, evidence of prior SARS-CoV-2 infection, count of pre-existing chronic conditions by organ system, presence of a positive PCR or antigen test, presence of a COVID-19 condition code, SARS-CoV-2 antiviral use, presence of comorbid respiratory conditions for which acute steroids are indicated (croup, asthma, bronchiolitis, cystic fibrosis exacerbation), prior healthcare utilization, evidence of obesity and presence of high-risk conditions. Detailed variable definitions are provided in S2 Table in S2 File. We defined chronic conditions by organ system using the Pediatric Medical Complexity Algorithm (PMCA) Version 3.0 [11], which counted the number of organ systems (17 total) in which diagnoses were recorded up to three years before T0. Healthcare utilization included all encounter types (inpatient, telehealth, outpatient, etc.). The distribution of the number of each visit type per patient within three years prior to cohort entry was analyzed and cut-off points were determined to create levels of utilization for each type of visit. All time-dependent covariates were measured on or prior to T0.

### Analyses

We applied principles of randomized controlled trials to emulate a target trial, following the two-step process outlined by Hernan et al. [12,13]. First, we developed the causal question of interest in the form of a hypothetical trial protocol. Second, we emulated each component of this protocol using observational EHR data. Target trial emulation specifications are included in **S1 Table**.

We applied inverse probability of treatment weighting (IPTW) for the average treatment effect among the treated (ATT) estimand to achieve covariate balance between treated and untreated participants. This approach emulated a clinical trial in which participants are allocated at random to a treatment or placebo group. A covariate was defined as balanced when an absolute value standardized mean difference (SMD) between groups was less than 0.2. Weights were considered extreme if they were >99.5^th^ or <0.5^th^ percentile and were redefined using the values of weights at the 99.5^th^ or 0.5^th^ percentile. Weighted incidence rates per 1,000 person-years were reported with bootstrapped 95% confidence intervals estimated based on Poisson distribution. Incidence rate differences between treated and untreated patients were calculated. Weighted Cox proportional hazards regression was used to compare the risk of outcomes between steroid recipients and non-recipients, with follow-up occurring within 1–6 months after the cohort entry date. Patients were right-censored at time of death, SARS-CoV-2 vaccination, remdesivir or nirmatrelvir/ritonavir use, or steroid use. We calculated hazard ratios from Cox proportional hazards models with 95% confidence intervals (after testing the proportional hazards assumption). All statistical tests were two-sided, with P-values below 0.05 deemed statistically significant. All data were analyzed in R version 4.4.0 in combination with the open-source packages *WeightIt, survey, survminer and ggpubr* [14–17]*.*

### Sensitivity analyses

We performed additional sensitivity analyses in which weighting and Cox regressions were re-computed within subgroups and strata of interest. Groups were stratified by age (<5 years; 5–12 years; ≥ 13 years), COVID era (pre-Omicron: before December 1, 2021; in-Omicron: on or after December 1, 2021) and healthcare utilization within the 7–365 days prior to the COVID date (0–1 visits; 2–6 visits; ≥ 7 visits). We also examined subgroups excluding steroid use within 1–180 days prior to the COVID infection date and excluding patients with comorbidities during acute SARS-CoV-2 infection (croup, asthma, bronchiolitis, cystic fibrosis exacerbation). Analyses were rerun limiting to patients who received dexamethasone, rather than the original list of steroids. Analyses were also rerun removing steroid use or SARS-CoV-2 vaccination during the follow-up period from the list of censoring reasons, to address concerns about potential informative censoring (e.g., patients who receive steroids during follow-up may be more likely to develop PASC but may be censored prior to development of PASC). In another sensitivity analysis, the weighting step included a variable indicating the PEDSnet acute COVID-19 severity definition, which categorizes patients into asymptomatic, mild, moderate or severe [18]. Analyses were considered exploratory and were not corrected for multiple comparisons.

## Results

### Cohort identification

Application of attrition criteria resulted in a cohort of 854,128 children/youth, of whom 768,845 (90.0%) were outpatients and 85,283 (10.0%) were inpatients at the time of SARS-CoV-2 infection. Amongst the hospitalized cohort, there were 11,250 children with SARS-CoV-2 infection who received steroids during their acute illness and 74,033 children (post-weighting: n = 10,340) with SARS-CoV-2 infection who did not receive steroids (S4 Table in S2 File). Amongst the outpatient cohort, there were 22,085 children with SARS-CoV-2 infection who received steroids during their acute illness and 746,760 children (post-weighting: n = 20,373) with SARS-CoV-2 infection who did not receive steroids (S4 Table in S2 File). Inverse probability of treatment weighting successfully balanced steroid-untreated and steroid-treated patients on all covariates within both the outpatient and hospitalized subgroups (S1-S4 Figs in S2 File).

Within the balanced hospitalized cohort, the mean age of patients was 6.4 years for treated patients and 6.6 years for untreated patients (**Table 1**). Both treated and untreated patients were more likely to be male and were more likely to be non-Hispanic white than any other race-ethnicity category (untreated: 40.3%; treated: 39.5%). Most treated and untreated patients had at least one high-risk condition (defined as high risk for developing SARS-CoV-2 complications) within the 3 years prior to their cohort entry date (untreated: 80.8%; treated: 81.0%). Among the balanced outpatient cohort, the mean age of treated patients was 6.8 years and the mean age of untreated patients was 7.4 years (**Table 2**). Similar to the hospitalized cohort, outpatient treated and untreated patients were more likely to be male and non-Hispanic White. Most treated and untreated patients had evidence of at least one high-risk condition within the 3 years prior to their cohort entry date (untreated: 62.8%; treated: 60.1%).

**Table 1:** Inverse-probability-of-treatment-weighted sociodemographic and clinical characteristics of the study population hospitalized during SARS-CoV-2 infection.

Characteristic	Not treated with steroids N = 10,340^1^	Treated with steroids N = 11,250^1^	Standardized Mean Difference^2^
Age at T0 (years) (Standard Deviation)	6.56 (6.15)	6.44 (6.06)	−0.02
Age Group at T0 (years)			
<1	2,314 (22.38%)	2,451 (21.79%)	−0.01
1–5	3,667 (35.47%)	4,194 (37.28%)	0.04
6–9	1,099 (10.63%)	1,220 (10.84%)	0.01
10–13	1,288 (12.46%)	1,318 (11.72%)	−0.02
14–19	1,971 (19.06%)	2,067 (18.37%)	−0.02
Sex			
Male or Other/unknown/ambiguous	5,929 (57.34%)	6,427 (57.13%)	0.00
Female	4,411 (42.66%)	4,823 (42.87%)	0.00
Race/Ethnicity			
Non-Hispanic White	4,169 (40.32%)	4,446 (39.52%)	−0.02
Hispanic	2,748 (26.58%)	3,056 (27.16%)	0.01
Non-Hispanic Asian	327 (3.16%)	362 (3.22%)	0.00
Non-Hispanic Black/African American	1,639 (15.85%)	1,782 (15.84%)	0.00
Non-Hispanic Other or Multiple Race	299 (2.89%)	325 (2.89%)	0.00
Other/Unknown	1,159 (11.21%)	1,279 (11.37%)	0.01
PMCA Count of Body Systems			
No body systems	3,828 (37.02%)	4,060 (36.09%)	−0.02
1 body system	2,725 (26.36%)	3,295 (29.29%)	0.06
2 body systems	1,228 (11.88%)	1,300 (11.56%)	−0.01
3-17 body systems	2,558 (24.74%)	2,595 (23.07%)	−0.04
Nirmatrelvir/ritonavir during acute period	26 (0.25%)	26 (0.23%)	0.00
Remdesivir during acute period	1,155 (11.17%)	1,948 (17.32%)	0.16
COVID diagnosis code during acute period	9,366 (90.58%)	10,262 (91.22%)	0.02
Positive PCR or antigen test during acute period	4,879 (47.18%)	5,584 (49.64%)	0.05
Number of visits in the 7–365 days prior to SARS-CoV-2 infection			
0 to 1 visits	3,872 (37.45%)	4,307 (38.28%)	0.02
2 to 6 visits	2,095 (20.26%)	2,329 (20.70%)	0.01
7 to 15 visits	1,535 (14.85%)	1,672 (14.86%)	0.00
16 + visits	2,838 (27.44%)	2,942 (26.15%)	−0.03
3-Month T0 Period			
Mar-May 2020	143 (1.38%)	126 (1.12%)	−0.03
Jun-Aug 2020	221 (2.14%)	223 (1.98%)	−0.01
Sep-Nov 2020	270 (2.61%)	294 (2.61%)	0.00
Dec-Feb 2021	457 (4.42%)	483 (4.29%)	−0.01
Mar-May 2021	415 (4.01%)	452 (4.02%)	0.00
Jun-Aug 2021	451 (4.36%)	565 (5.02%)	0.03
Sep-Nov 2021	721 (6.97%)	868 (7.72%)	0.03
Dec-Feb 2022	1,871 (18.09%)	2,105 (18.71%)	0.02
Mar-May 2022	746 (7.21%)	770 (6.84%)	−0.01
Jun-Aug 2022	1,166 (11.28%)	1,227 (10.91%)	−0.01
Sep-Nov 2022	848 (8.20%)	912 (8.11%)	0.00
Dec-Feb 2023	909 (8.79%)	973 (8.65%)	−0.01
Mar-May 2023	450 (4.35%)	462 (4.11%)	−0.01
Jun-Aug 2023	234 (2.26%)	249 (2.21%)	0.00
Sep-Nov 2023	604 (5.84%)	665 (5.91%)	0.00
Dec-Feb 2024	756 (7.31%)	796 (7.08%)	−0.01
Mar-May 2024	78 (0.75%)	80 (0.71%)	0.00
Initial SARS-CoV-2 series (2 within 56 days, with second at least 14 days prior to COVID date)	855 (8.27%)	860 (7.64%)	−0.02
Diagnoses during the acute period (asthma, croup, bronchiolitis, cystic fibrosis exacerbation)	5,221 (50.50%)	5,894 (52.39%)	0.04
Measured obesity within 1.5 years prior to T0 date	1,477 (14.28%)	1,736 (15.43%)	0.03
Has 1 or more high-risk conditions within 3 years prior to T0 date	8,355 (80.80%)	9,112 (81.00%)	0.00
Prior COVID infection (at least 60 days prior to COVID date)	940 (9.09%)	993 (8.83%)	−0.01
Steroid Route			
Oral	NA	5,998 (53.32%)	NA
Intravenous Injection	NA	4,658 (41.40%)	NA
Oral and Intravenous Injection	NA	594 (5.28%)	NA
Mean visits 28–179 days after T0 (Standard Deviation)	8.05 (13.33)	8.01 (13.92)	0.00
Mean visits 180–365 days after T0 (Standard Deviation)	7.83 (13.86)	7.24 (14.17)	−0.05

^1^categorical variables: n (%); continuous variables: mean (standard deviation).

^2^Standardized Mean Differences were computed following IPTW and the redefinition of extreme weights.

Abbreviations: PMCA- pediatric medical complexity algorithm, PCR- polymerase chain reaction

**Table 2:** Inverse-probability-of-treatment-weighted sociodemographic and clinical characteristics of the study population not hospitalized during SARS-CoV-2 infection.

Characteristic	Untreated, N = 20,373^1^	Steroid-Treated, N = 22,085^1^	Standardized Mean Difference^2^
Age at T0 (years) (Standard Deviation)	7.41 (6.11)	6.84 (6.01)	−0.09
Age Group at T0 (years)			
<1	3,389 (16.63%)	3,592 (16.26%)	−0.01
1–5	7,127 (34.98%)	8,830 (39.98%)	0.10
6–9	2,746 (13.48%)	2,867 (12.98%)	−0.01
10–13	2,603 (12.78%)	2,544 (11.52%)	−0.04
14–19	4,508 (22.13%)	4,252 (19.25%)	−0.07
Sex			
Male or Other/unknown/ambiguous	11,509 (56.49%)	12,677 (57.40%)	−0.02
Female	8,864 (43.51%)	9,408 (42.60%)	−0.02
Race/Ethnicity			
Non-Hispanic White	7,604 (37.32%)	8,278 (37.48%)	0.00
Hispanic	5,010 (24.59%)	5,529 (25.04%)	0.01
Non-Hispanic Asian	582 (2.86%)	671 (3.04%)	0.01
Non-Hispanic Black/African American	2,999 (14.72%)	3,024 (13.69%)	−0.03
Non-Hispanic Other or Multiple Race	405 (1.99%)	440 (1.99%)	0.00
Other/Unknown	3,774 (18.52%)	4,143 (18.76%)	0.01
PMCA Count of Body Systems			
No body systems	11,622 (57.05%)	13,116 (59.39%)	0.05
01 body system	5,727 (28.11%)	5,981 (27.08%)	−0.02
02 body systems	1,807 (8.87%)	1,785 (8.08%)	−0.03
03-17 body systems	1,217 (5.97%)	1,203 (5.45%)	−0.02
Nirmatrelvir/ritonavir during acute period	211 (1.04%)	192 (0.87%)	−0.02
Remdesivir during acute period	41 (0.20%)	56 (0.25%)	0.01
COVID diagnosis code during acute period	18,104 (88.86%)	19,644 (88.95%)	0.00
Positive PCR or antigen test during acute period	10,029 (49.22%)	11,377 (51.51%)	0.05
Number of visits in the 7–365 days prior to SARS-CoV-2 infection			
0 to 1 visits	7,396 (36.30%)	8,296 (37.56%)	0.03
2 to 6 visits	5,943 (29.17%)	6,367 (28.83%)	−0.01
7 to 15 visits	3,956 (19.42%)	4,163 (18.85%)	−0.01
16 + visits	3,079 (15.11%)	3,259 (14.76%)	−0.01
3-Month T0 Period			
Mar-May 2020	84 (0.41%)	83 (0.38%)	−0.01
Jun-Aug 2020	283 (1.39%)	280 (1.27%)	−0.01
Sep-Nov 2020	413 (2.03%)	415 (1.88%)	−0.01
Dec-Feb 2021	699 (3.43%)	710 (3.21%)	−0.01
Mar-May 2021	584 (2.87%)	578 (2.62%)	−0.02
Jun-Aug 2021	901 (4.42%)	886 (4.01%)	−0.02
Sep-Nov 2021	1,293 (6.35%)	1,291 (5.85%)	−0.02
Dec-Feb 2022	4,940 (24.15%)	5,487 (24.84%)	0.02
Mar-May 2022	1,471 (7.22%)	1,628 (7.37%)	0.01
Jun-Aug 2022	2,553 (12.53%)	2,903 (13.14%)	0.02
Sep-Nov 2022	1,949 (9.56%)	2,063 (9.34%)	−0.01
Dec-Feb 2023	1,770 (8.69%)	1,936 (8.77%)	0.00
Mar-May 2023	583 (2.86%)	651 (2.95%)	0.01
Jun-Aug 2023	648 (3.18%)	732 (3.31%)	0.01
Sep-Nov 2023	1,087 (5.33%)	1,235 (5.59%)	0.01
Dec-Feb 2024	1,059 (5.20%)	1,122 (5.08%)	−0.01
Mar-May 2024	77 (0.38%)	85 (0.38%)	0.00
Initial SARS-CoV-2 vaccine series (2 within 56 days, with second at least 14 days prior to COVID date	2,003 (9.83%)	2,019 (9.14%)	−0.02
Comorbidities during the acute period (asthma, croup, bronchiolitis, cystic fibrosis exacerbation)	10,201 (50.07%)	11,919 (53.97%)	0.08
Measured obesity within 1.5 years prior to T0 date	3,066 (15.05%)	3,038 (13.76%)	−0.04
Has 1 or more high-risk conditions within 3 years prior to T0 date	12,799 (62.82%)	13,280 (60.13%)	−0.06
Prior COVID infection (at least 60 days prior to COVID date)	1,351 (6.63%)	1,626 (7.36%)	0.03
Steroid Route			
Oral	NA	7,801 (35.32%)	NA
Intravenous Injection	NA	12,967 (58.71%)	NA
Oral and Intravenous Injection	NA	1,317 (5.96%)	NA
Visits 28–179 days after T0	2.98 (4.83)	2.91 (5.04)	−0.02
Visits 180–365 days after T0	3.03 (5.15)	2.84 (5.24)	−0.04

^1^categorical variables: n (%); continuous variables: mean (standard deviation).

^2^Standardized Mean Differences were computed following IPTW and the redefinition of extreme weights.

Abbreviations: PMCA- pediatric medical complexity algorithm, PCR- polymerase chain reaction

### Primary and secondary analyses

Within the hospitalized sub-group, weighted incidence rates of the PASC computable phenotype were 94.97 per 1,000 person-years within treated patients and 99.61 per 1,000 person-years within untreated patients. In outpatients, weighted incidence rates of the PASC computable phenotype were 40.66 per 1,000 person-years in treated patients and 39.39 per 1,000 person-years in untreated patients (**Table 3**).

**Table 3:** Weighted 1-6 month incidence rates between treatment and non-treatment group in outpatient and hospitalized settings.

Group	PASC outcome	Untreated rate per 1,000 person-years	Treated rate per 1,000 person-years	Treated – Untreated Rate Difference (95% CI)
**Hospitalized**	Computable phenotype	99.61 (90.79, 109.27)	94.97 (86.61, 104.14)	−4.64 (−17.07, 7.73)
	U09.9 Diagnosis code	3.02 (1.79, 5.11)	8.04 (5.87, 11.00)	5.02 (2.43, 7.92)
	Respiratory	24.82 (20.65, 29.83)	26.68 (22.45, 31.70)	1.86 (−4.13, 7.83)
	Neurological/POTS	13.87 (10.85, 17.73)	13.42 (10.52, 17.11)	−0.45 (−5.11, 3.69)
	Gastrointestinal	13.40 (10.44, 17.21)	7.83 (5.70, 10.76)	−5.57 (−9.38, −1.76)
	Musculoskeletal	10.32 (7.76, 13.72)	10.52 (8.00, 13.85)	0.20 (−3.53, 3.99)
**Outpatient**	Computable phenotype	39.39 (35.58, 43.62)	40.66 (36.89, 44.81)	1.27 (−3.36, 6.01)
	U09.9 Diagnosis code	1.85 (1.16, 2.96)	1.98 (1.28, 3.07)	0.13 (−0.84, 1.17)
	Respiratory	12.21 (10.17, 14.65)	14.12 (11.98, 16.64)	1.91 (−0.66, 4.68)
	Neurological/POTS	6.95 (5.46, 8.85)	8.05 (6.47, 10.00)	1.10 (−0.82, 3.18)
	Gastrointestinal	5.23 (3.96, 6.91)	4.76 (3.59, 6.32)	−0.47 (−2.14, 1.32)
	Musculoskeletal	5.27 (3.99, 6.95)	5.16 (3.93, 6.77)	−0.11 (−1.78, 1.61)

**Fig 1:**
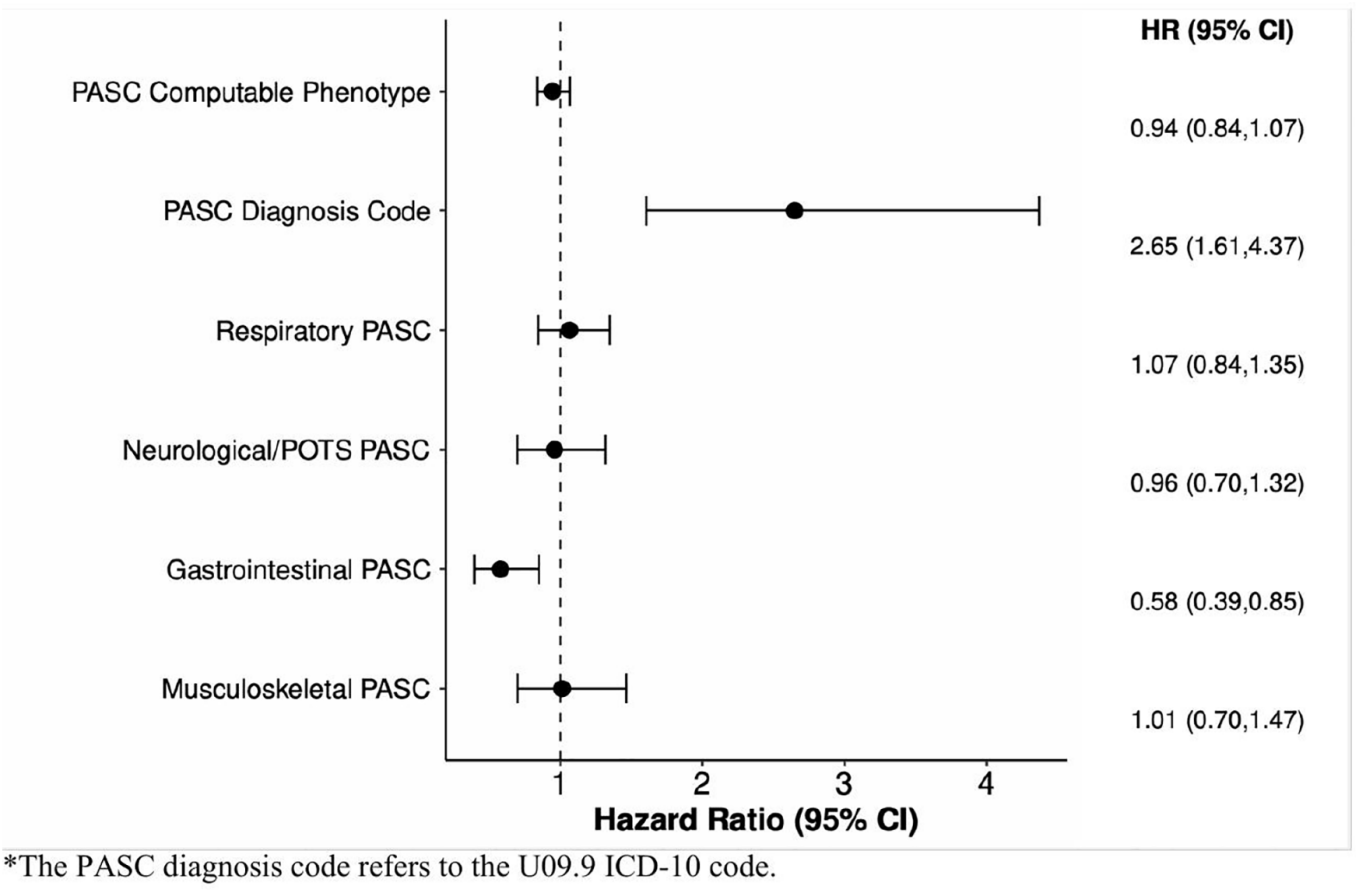
Cox proportional hazard models comparing the steroid treated group versus the untreated group in the development of PASC in the 1-6 months following acute infection within the hospitalized cohort. The PASC diagnosis code refers to the U09.9 ICD-10 code.

**Fig 2:**
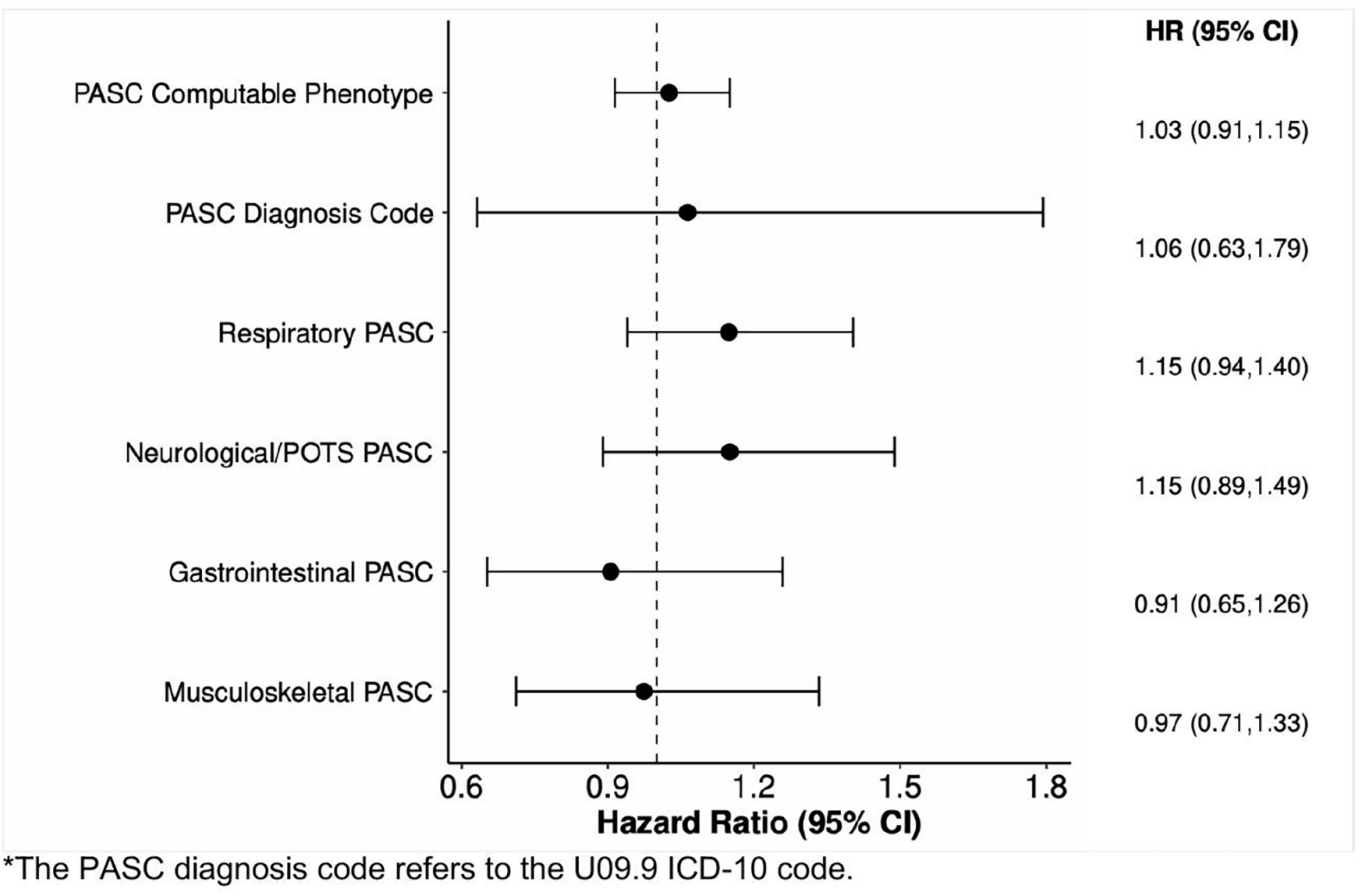
Cox proportional hazard models comparing the steroid treated group versus the untreated group in the development of PASC in the 1-6 months following acute infection within the outpatient cohort. The PASC diagnosis code refers to the U09.9 ICD-10 code.

The proportional hazards assumption was met for all Cox models (p > 0.05). In the cohort hospitalized during their acute SARS-CoV-2 infection, there was no difference observed in the development of PASC in the subsequent 1–6 months following acute SARS-CoV-2 infection between the group receiving acute steroids and the group who did not receive steroids. No differences were observed for PASC subphenotypes including children/youth with respiratory, neurologic, and musculoskeletal presentations. There was a lower risk of gastrointestinal manifestations of PASC in the group who received steroids compared with those who did not receive steroids (HR: 0.58; [95% CI: 0.39–0.85], p = 0.01) (**Fig 1** and S5 Fig in S2 File). These secondary PASC subphenotype outcomes were exploratory; after Bonferroni correction (a = 0.00625), the reduced risk of GI PASC was not significant. Further exploration of the GI sub-phenotype outcome did not demonstrate a specific treatment effect among those with pre-existing GI conditions (S5 Table in S2 File). Children receiving steroids were more likely to receive a U09.9 ICD-10 diagnosis code for PASC compared with those not receiving steroids (HR: 2.65; 95% CI: [1.61–4.37], p < 0.01). In the outpatient subgroup, there was also no difference in the development of PASC in the subsequent 1–6 months following acute SARS-CoV-2 infection between the group receiving acute steroids and the group who did not receive steroids. Similarly, no differences were observed for the U09.9 ICD-10 diagnosis code for PASC or for PASC subphenotypes including respiratory, neurologic, gastrointestinal and musculoskeletal (**Fig 2** and S6 Fig in S2 File).

### Sensitivity analyses

In patients hospitalized during COVID with high prior utilization (≥ 7 visits within the year prior to SARS-CoV-2 infection), steroid-treated patients had a reduced risk of the PASC computable phenotype (HR: 0.81; [95% CI: 0.70–0.95], p = 0.01). Stratification by age group revealed an increased risk of the PASC computable phenotype in outpatients aged 13 and older who were treated with steroids (HR: 1.29; [95% CI: 1.08–1.55], p = 0.01) (S6 Table in S2 File). An increased risk of the PASC computable phenotype was also observed in outpatients with no acute comorbidities during SARS-CoV-2 infection who were treated, compared with untreated patients (HR: 1.23; [95% CI: 1.08–1.42], p < 0.01). No differences in risk of the PASC computable phenotype were observed within stratifications by COVID era or underlying gastrointestinal condition, when excluding steroid use within 180 days prior to COVID, or when including the PEDSnet variable for SARS-CoV-2 severity in weighting.

When removing steroid use and SARS-CoV-2 vaccination as reasons for censoring, an increased risk of the U09.9 code was observed for steroid-treated patients within the hospitalized cohort (HR: 2.83; [95% CI: 1.78–4.48], p < 0.01) but the risk of gastrointestinal PASC was no longer significantly lower in treated patients (**S7 Fig in** S2 File). In the outpatient cohort, treatment effects were similar to those of the main analysis, with no differences observed (**S8 Fig in** S2 File). When redefining treatment to include only patients who received dexamethasone, an increased risk of the U09.9 code was observed for treated patients compared with untreated patients in the hospitalized cohort (HR: 2.42; [95% CI: 1.46–4.00], p < 0.01) and no treatment effects were observed within the outpatient cohort (**S9 and S10 Figs** in S2 File).

## Discussion

In our retrospective target trial emulation evaluating the impact of steroids during the acute phase of COVID infection in children and youth, we did not find a decreased risk of PASC in children receiving steroids compared with those who did not receive steroids during the acute infectious period in inpatient and outpatient settings. These findings warrant further exploration of specific steroid regimens in the prevention of PASC.

Acute SARS-CoV-2 infection is characterized by substantial immune dysregulation, particularly among severe cases, and anti-inflammatory therapy is recommended for individuals who meet criteria for severe disease [19]. There has been some suggestion that the use of immunomodulators including steroids during the acute phase of SARS-CoV-2 infection may be beneficial in the prevention of long COVID [20]. The glucocorticoid receptor acts as a transcription regulatory factor and represses the expression of inflammatory cytokines, chemokines, and prostaglandins, suppresses the antigen-stimulated inflammation mediated by macrophages, dendritic cells, and epithelial cells, and impairs cytotoxic immune responses by downregulating interferon-γ production and inhibiting the development of type-1 helper T cells, CD8 + T cells, and natural killer cells [21]. In a case-control observational study, patients who received oral dexamethasone for hospitalized COVID-19 were less likely to experience persistent symptoms at 8-month follow-up [22]. Decreased levels of cortisol and cortisone have been observed to be associated with PASC [23]. Further data also suggest decreased levels of cortisol among individuals with long COVID [24]. However, we did not observe benefits from steroids during acute COVID for most manifestations of PASC in our study of children and youth except for a decreased risk of GI manifestations of long COVID. While there is some biologic plausibility for this (in vitro studies suggest that SARS-CoV-2 infection impacts the GI epithelium, inducing an inflammatory and anti-viral response, [25] with the production and release of cytokines, [26] and a complex interplay with the gut microbiota, further contributing to inflammation and dysregulated immune responses in the GI tract [27]), this finding requires further study through mechanistic studies and larger prospective trials.

In the group of children and youth who were hospitalized for their acute COVID infection, we observed an increased risk of the PASC diagnosis code for steroid-treated patients compared with untreated patients. This finding may reflect detection bias, in which sicker patients are treated with steroids during the acute infection and are also more likely to be evaluated in subsequent follow-up visits, or have symptoms attributed to long COVID, and thus are more likely to be coded as PASC. Weighting may not have fully balanced groups on unmeasured confounders, and groups may still have differed by complexity and disease chronicity. Similarly, there was a non-significant increase in the incidence of neurologic and respiratory PASC in the treated outpatient group, which also may reflect medical complexity and different health-seeking patterns that weren’t completely adjusted for in our IPTW.

Sensitivity analyses identified an increased risk of PASC in steroid-treated outpatients aged 13 or older, and in treated outpatients who did not have evidence of acute comorbidities during COVID (asthma, bronchiolitis, croup, or cystic fibrosis exacerbation), compared with untreated outpatients. The potential for detection bias and increased follow-up in treated patients should be noted. A decreased risk of PASC was observed in treated patients hospitalized during COVID with high prior utilization, compared with untreated patients. It is important to note that sensitivity analyses are exploratory and multiple comparison correction has not been implemented; further investigation is needed to understand the effects of steroid use within these populations.

This study has several strengths. To our knowledge, this study is the first to evaluate the benefit of steroids during acute COVID in the prevention of long COVID in children. The use of comprehensive sociodemographic, clinical, and geocoding inpatient and outpatient data from EHR data sources across the US confers high population representativeness, and the adoption of a target trial emulation approach (including the specification of the hypothetical randomized pragmatic trial) helped mitigate many of the typical challenges and biases in observational data analyses, including selection bias, immortal time bias and confounding by indication. Observational studies can provide evidence on the effectiveness of interventions. However, several limitations are worth noting. Firstly, decreased health system-based testing during the omicron period and increased steroid use for hospitalized patients may bias our population to sicker individuals. Next, variability in steroid type, dosing and duration may confer different treatment effects; however, these data were not consistently coded in the EHR to enable examination. While the analytic approach attempts to mitigate bias, confounding by indication remains possible and children who received steroids may have been sicker than untreated patients, which could affect their risk of long COVID. Prior steroid exposure was defined as children with steroid use occurring within 3 months prior to SARS-CoV-2 infection and may not exclude all patients with chronic steroid use who did not have healthcare encounters within this period, potentially resulting in a sicker cohort of patients than those included in a traditional clinical trial. Our computable phenotype has decreased performance for youth with underlying comorbidities, which can lead to long COVID misclassification. While we attempted to account for time of infection through weighting and stratified sensitivity analyses, the study spans multiple COVID-19 periods and includes substantial changes in variants, vaccination status, U09.9 adoption timing and clinical practices. Finally, our study may fail to account for other confounders such as organ system(s) involved and degree of immune activation in acute COVID and treatments used to alleviate symptoms of long COVID.

### Conclusion

In our target trial emulation of children with COVID, the use of steroids in the acute illness phase among those who were inpatients and outpatients at the time of SARS-CoV-2 infection was not shown to impact the development of long COVID symptoms in the 1–6 months following infection. Additional studies are needed to enhance our understanding of their benefit in the prevention of long COVID.

## Supporting information

S1 Table RECOVER-EHR Consortium members. (DOCX)

S2 File Supplement. Supporting information for S2-S6 Tables and S1-S10 Figs. (DOCX)

## Abbreviations

COVID-19: coronavirus disease 2019
SARS-CoV-2: severe acute respiratory syndrome coronavirus
PCR: polymerase chain reaction
EHR: electronic health record
CDC: Centers for Disease Control and Prevention
OR: odds ratios
ED: emergency department
UC: Urgent Care
CI: confidence intervals
ICD-10: International Classification of Diseases, version 10
