# Supplementary material for: Evaluation of steroids for acute COVID in the prevention of long COVID in children: An EHR and pediatric cohort study from the RECOVER Initiative
Source: PLoS One. 2026 Jun 18;21(6):e0350888. doi: 10.1371/journal.pone.0350888 (PMC13278399; doi:10.1371/journal.pone.0350888)
Supplement: S1 Table — (DOCX) [file pone.0350888.s001.docx]

# RECOVER-EHR Consortium Members

## Study Group Leads

Ivan Diaz

[ivan.diaz@nyulangone.org](mailto:ivan.diaz@nyulangone.org)

Rachel Kenny

[Rachel.kenny@nyulangone.org](mailto:Rachel.kenny@nyulangone.org)

EHR Clinical Science Core

New York University

*Iván Diaz, PI*

*Rachel Kenny, PI*

Jasmin Divers^

Lorna Thorpe^

Hannah Mandel

Jennifer Truong

Shannon Wilneff Wuller

**PCORnet Core Contributors**

**Louisiana Public Health Institute:** *Tom Carton, mPI,* Anna Legrand, Elizabeth Nauman

**Weill Cornell Medicine:** *Rainu Kaushal, mPI, Mark Weiner, mPI,*

**Children’s Hospital of Philadelphia:** *L. Charles Bailey, mPI, Christopher B. Forrest, mPI,*

**Children’s Hospital of Colorado:** *Suchitra Rao, mPI*

Data Contributors

Albert Einstein College of Medicine

Selvin Soby

Ann & Robert H. Lurie Children's Hospital of Chicago

*Ravi Jhaveri, PI*

Children's Hospital of Philadelphia

*L. Charles Bailey, mPI*

*Christopher B. Forrest, mPI*

Children's National Hospital

Dongkyu Kim

Cincinnati Children's Hospital Medical Center

*Nathan M. Pajor, PI*

Jyothi Priya Alekapatti Nandagopal

Columbia University

*Soumitra Sengupta, PI*

Duke University Health System

*W. Schuyler Jones, PI*

Curtis A. Kieler

Emory University

Nita N. Deshpande

Feinberg School of Medicine, Northwestern University

*David Liebovitz, PI*

Icahn School of Medicine at Mount Sinai

*Carol Horowitz, PI*

Intermountain Healthcare

Heidi T. May

Intermountain Medical Center Heart Institute

*Stacey Knight, PI*

Louisiana Public Health Institute

*Thomas W. Carton, PI*

Medical College of Wisconsin

*Bradley Taylor, PI*

Alex Stoddard

Nationwide Children's Hospital

*Kelly Kelleher, PI*

Yungui Huang

Nemours/Alfred I. duPont Hospital for Children

*H. Timothy Bunnell, PI*

**Nicklaus Children's Hospital**

*Sandy L. Gonzalez, PI*

Maurice Duque

New York University Langone Health

*Saul Blecker, PI*

Nathalia Ladino

OCHIN, Inc.

*Wyatt Bensken, PI*

Ochsner Health System

*Dan Fort, PI*

Penn State U College of Medicine

Wenke Hwang, PI

Seattle Children's Hospital

*Dimitri Christakis, PI*

Daksha Ranade

Stanford University School of Medicine

Keith E. Morse

Temple University

*Dan Rubin, PI*

John Turella

The Ohio State University

*Soledad Fernandez, PI*

Neena Thomas

The Research Institute at Nationwide Children’s Hospital

Yungui Huang

University Medical Center New Orleans

*Yuriy Bisyuk, PI*

University of California San Francisco

*Susan Kim, PI*

Mark Pletcher

University of Colorado School of Medicine and Children’s Hospital Colorado

*Suchitra Rao, PI*

Sara J. Deakyne Davies

University of Florida

*Mei Lui, PI*

Jiang Bian

University of Iowa

*Elizabeth Chrischilles, PI*

University of Michigan

*David Williams, PI*

University of Missouri School of Medicine

*Xing Song, PI*

University of Nebraska Medical Center

*Carol Geary, PI*

Jim Svoboda

University of Pittsburgh

*Jonathan Arnold, PI*

*Michael Becich, PI*

*Yalini Senathirajah, PI*

Nickie Cappella

University of Utah

*Mollie R. Cummins, PI*

Ramkiran Gouripeddi

Vanderbilt University Medical Center

Yacob Tedla, PI

Wei-Qi Wei

Wake Forest School of Medicine

*Stephen M. Downs, PI*

Brian Ostasiewski

Weill Cornell Medicine

*Rainu Kaushal, PI*

Thomas Campion
