# Supplementary material for: Evaluation of steroids for acute COVID in the prevention of long COVID in children: An EHR and pediatric cohort study from the RECOVER Initiative
Source: PLoS One. 2026 Jun 18;21(6):e0350888. doi: 10.1371/journal.pone.0350888 (PMC13278399; doi:10.1371/journal.pone.0350888)
Supplement: S2 File — (DOCX) [file pone.0350888.s002.docx]

**Supporting Information**

| **Table/Figure** | **Page** |
| --- | --- |
| **S1 Table.** RECOVER-EHR Consortium members. | 3 |
| **S2 Table.** Target trial emulation specification definitions of eligibility criteria, treatments, and outcomes. | 4 |
| **S3 Table.** Detailed cohort inclusion criteria. | 6 |
| **S4 Table.** Attrition table indicating final derived cohort. | 9 |
| **S1 Fig.** Love plot displaying standardized mean differences before and after applying inverse probability of treatment weighting (IPTW) in children and youth hospitalized with SARS-CoV-2 infection. | 10 |
| **S2 Fig.** Density plot of post-truncated weights in treated and untreated groups among children and youth hospitalized with SARS-CoV-2 infection. | 11 |
| **S3 Fig.** Love plot displaying standardized mean differences before and after applying inverse probability of treatment weighting (IPTW) in children and youth outpatients with SARS-CoV-2 infection. | 12 |
| **S4 Fig.** Density plot of post-truncated weights in treated and untreated groups among children and youth outpatients with SARS-CoV-2 infection. | 13 |
| **S5 Fig.** Cumulative incidence curves comparing the weighted cumulative incidence of PASC between the treated and untreated groups among children and youth hospitalized with SARS-CoV-2 infection. | 14 |
| **S6 Fig.** Cumulative incidence curves comparing the weighted cumulative incidence of PASC between the treated and untreated groups among children and youth outpatients with SARS-CoV-2 infection | 15 |
| **S5 Table.** Hazard Ratios from Cox proportional hazard models comparing risk of gastrointestinal PASC between the treated and untreated groups among children and youth with SARS-CoV-2 infection stratified by underlying gastrointestinal condition | 16 |
| **S6 Table.** Hazard Ratios from Cox proportional hazard models comparing risk of the PASC computable phenotype between the treated and untreated groups (hospitalized and outpatient) among children and youth with SARS-CoV-2 infection stratified by underlying gastrointestinal condition, age, COVID era, utilization within the year prior to the COVID date, excluding patients with steroid use within 180 days prior to COVID infection, and excluding patients with acute comorbidities. | 17 |
| **S7 Fig.** Cox proportional hazard models comparing the steroid-treated group versus the untreated group in the development of PASC in the 1-6 months following acute infection within the hospitalized cohort, removing steroid use during follow-up and SARS-CoV-2 immunization during follow-up as censoring reasons. | 19 |
| **S8 Fig.** Cox proportional hazard models comparing the steroid-treated group versus the untreated group in the development of PASC in the 1-6 months following acute infection within the outpatient cohort, removing steroid use during follow-up and SARS-CoV-2 immunization during follow-up as censoring reasons. | 20 |
| **S9 Fig.** Cox proportional hazard models comparing the steroid-treated group versus the untreated group in the development of PASC in the 1-6 months following acute infection within the hospitalized cohort, limiting to patients with dexamethasone use. | 21 |
| **S10 Fig.** Cox proportional hazard models comparing the steroid group versus the untreated group in the development of PASC in the 1-6 months following acute infection within the outpatient cohort, limiting to patients with dexamethasone use. | 22 |

**S1 Table. RECOVER-EHR Consortium Members.**

(See full REOVER Consortium supplement.)

**S2 Table. Target Trial Emulation Specifications- Definitions of Eligibility criteria, Treatments, and Outcomes.**

|  | Target Trial Specification | Target Trial Emulation |
| --- | --- | --- |
| Eligibility criteria | Inclusion:  Age < 19 yrs at the time of SARS-CoV-2 infection (3/1/2020 to present)  COVID-positive (known infection date):  Positive PCR/antigen test  Positive COVID-specific diagnosis  Remdesivir/Nirmatrelvir/ritonavir exposure  Exclusion:  Patients who received steroids within 90 days prior to COVID index date (i.e. any course of steroids extending into the –90 to -1-day window)  Patients who initiated steroid treatment from days 12 to 28 following cohort entry date  Right censoring:  New positive test in the 1-6 months following acute infection  New steroid course or COVID vaccine or COVID antiviral (remdesivir; nirmatrelvir/ritonavir) in the 1-6 months following infection  Death during evaluation period | Same as target trial |
| Treatment strategies | Steroids for 1-10 days (oral or IV dexamethasone, prednisone, prednisolone, methylprednisolone) during acute COVID | Treated: Steroids for 1-10 days (dexamethasone, prednisone, prednisolone, methylprednisolone) during acute COVID  Untreated: No steroids during acute COVID |
| T0 | Date of randomization | Treated: Date of earliest steroid prescription within the 0-to-12-day window following COVID infection  Untreated: Date following COVID infection within the 0-to-12-day window. The distribution of the number of days between COVID infection date and the T0 date for untreated patients will be matched to the distribution of the number of days between COVID infection date and treatment initiation for treated patients. |
| Assignment procedures | Random assignment to steroid treatment arm or placebo arm | IPTW to balance covariates in both groups |
| Follow up | 1-6 months following acute COVID infection | 1-6 months following acute COVID infection |
| Outcomes | Development of any long COVID symptom in the 1-6 months after acute COVID infection (self-report) | Occurrence of long COVID in the 1-6 months following COVID infection based on computable phenotype definition |
| Secondary outcomes | Respiratory PASC  MSK PASC  GI PASC  U09.9 PASC  Neurologic/POTS/  constitutional symptoms | Respiratory PASC  MSK PASC  GI PASC  U09.9 PASC  Neurologic/POTS/  constitutional symptoms |
| Causal contrasts of interest | Intention to treat effect | Intention to treat effect |
| Strata of interest | Hospitalized at time of COVID infection  Outpatient at time of COVID infection | Hospitalized at time of COVID infection  Outpatient at time of COVID infection |

**S3 Table. Detailed cohort inclusion criteria.**

| **Cohort inclusion step** | **Description** |
| --- | --- |
| 1: Identify COVID records | Identify all records where a patient had evidence of COVID. |
| 2: Identify steroid exposure windows | Identify windows of steroid exposure in relation to COVID dates, including:   - Prior exposure (-1 to -90 days) - During acute COVID (+0 to +12 days, with a duration of 1 to 10 days) - In the post-acute window prior to follow-up (+13 to +27 days) - In the follow-up period (+28 to +179 days, or 1-6 months) |
| 3: Remove records with prior steroid exposures and long steroid durations | Remove records with exclusions occurring prior to patient COVID dates, including:   - Prior steroid exposure (-1 to –90 days) - Drug durations lasting greater than 10 days that began within the (+0 to +12-day) window (to avoid capture of steroids for non-acute COVID indications) |
| 4: Identify COVID dates for treated and untreated patients | **Treated patients:** Select a random date of COVID for patients who had steroids during their (+0 to +12-day) acute COVID period around cohort entry date)  **Untreated patients (control):** Identify patients with no evidence of steroids during their (+0 to +12-day) acute COVID period around cohort entry date  Remove patients with negative age at time of COVID cohort entry date |
| 5. Identify T0 dates for treated and untreated patients | **Treated patients:** Identify number of days between date of COVID and earliest date of steroid exposure within the (+0 to +12-day) treatment window. T0 is the treatment initiation date.  **Untreated patients (control):** Mirror the difference in the number of days between COVID and steroid treatment in the treated patients to generate a T0 date for the control group. |
| 6: Apply additional exclusions occurring prior to the 28-179-day follow-up period after COVID index date | Apply additional patient exclusions:   - PASC prior to the cohort entry date   Patients with any of the following events occurring during the post-acute period prior to follow-up (<28 days after cohort entry date):   - Death - SARS-CoV-2 vaccination - Steroid use - Remdesivir or Nirmatrelvir/ritonavir use - MIS-C |
| 7: Perform IPTW | Stratify groups by hospitalization status during COVID (hospitalized; outpatient)  Perform Inverse Probability of Treatment Weighting (IPTW) to balance treated and untreated patients. Cap extreme weights (>99.5th or <0.5th percentile) at the 99.5th or 0.5th percentile, respectively  Remove patients with evidence of the PASC computable phenotype within 1 to 27 days after cohort entry date.  Variables that are weighted on include: (Time invariant)   - Site - Sex - Race/ethnicity   (Based on COVID index date)   - Prior COVID (at least 60 days prior to COVID date) - Acute Nirmatrelvir/ritonavir (0 to 12 days after COVID date) - Acute remdesivir (0 to 12 days after COVID date) - Acute COVID diagnosis (0 to 12 days after COVID date) - Acute comorbidities: croup, asthma, bronchiolitis, cystic fibrosis exacerbation (0 to 12 days after COVID date) - Number of visits (within 7 to 365 days prior to COVID date) - Prior immunization (at least 2 vaccinations at least 16 days apart, occurring at least 14 days prior to COVID date)   (Based on T0 date)   - 3-month period of T0 date - Age group at T0 - Pediatric Medical Complexity Algorithm version 3.0: number of body systems (within 1096 days prior to T0 date) - Measured obesity (within 545 days prior to T0 date) - Any high-risk conditions (within 1096 days prior to T0 date)   **Note on missing data:** Variables display presence of conditions of interest (e.g., presence of a measured body mass index indicating obesity). Patients with no records of the conditions of interest were not labelled “missing”; these records were simply not included in indication of the condition. |
| 8: Identify follow-up times for statistical analysis | Exclude patients who become ineligible prior to first day of PASC eligibility (28 days after the COVID date). Start follow-up based on the COVID date (Day 1 = the day after the COVID date). Patients will be followed up until the earliest of the following events:   - Death - End of the follow-up period (179 days after the COVID index date) - The outcome of interest (PASC) - Receipt of steroids within 28-179 days after COVID - Receipt of a SARS-CoV-2 immunization within 28-179 days after COVID - Receipt of remdesivir or Nirmatrelvir/ritonavir within 28-179 days after COVID |

**S4 Table. Attrition table indicating final derived cohort.**

| **Attrition Step** | **Patient Count** |
| --- | --- |
| 1. Patients with a known COVID infection date during the study period, aged <19 at date of COVID, within eligible sites | 920,547 |
| 2a. Exclude: patients with prior chronic steroid exposure (-1 to -90 days before COVID) | 32,957 |
| 2b. Exclude: patients with drug durations >10 days starting in the acute COVID window (0 to +12 days) | 3,221 |
| 3. Patients without any of the above exclusions | 881,604 |
| 4a. Exclude: patients with MIS-C diagnosed within a patient’s follow-up period or earlier | 1,737 |
| 4b. Exclude: patients with prior PASC | 6,119 |
| 5. Patients without any of the above exclusions | 875,289 |
| 6a. Exclude post-acute events: patients who died within 27 days after COVID cohort entry date | 362 |
| 6b. Exclude post-acute events: patients who had a COVID vaccination within 0 to 27 days after the COVID cohort entry date | 16,104 |
| 6c. Exclude post-acute events: patients with remdesivir or Nirmatrelvir/ritonavir use 13 to 27 days after the COVID cohort entry date | 38 |
| 6d. Exclude post-acute events: patients with post-acute steroids in days 13-27 before follow-up | 4,763 |
| 7. Final cohort (prior to weighting) | 854,128 |
| 7a. Hospitalized during COVID cohort entry date | 85,283 |
| 7a.1. Steroid-treated, hospitalized during COVID cohort entry date | 11,250 |
| 7a.2. Steroid-untreated, hospitalized during COVID cohort entry date | 74,033 |
| 7b. Outpatient during COVID cohort entry date | 768,845 |
| 7b.1. Steroid-treated, outpatient during COVID cohort entry date | 22,085 |
| 7b.2. Steroid-untreated, outpatient during COVID cohort entry date | 746,760 |

**S1 Fig. Love plot displaying standardized mean differences before and after applying inverse probability of treatment weighting (IPTW) in children and youth hospitalized with SARS-CoV-2 infection.**


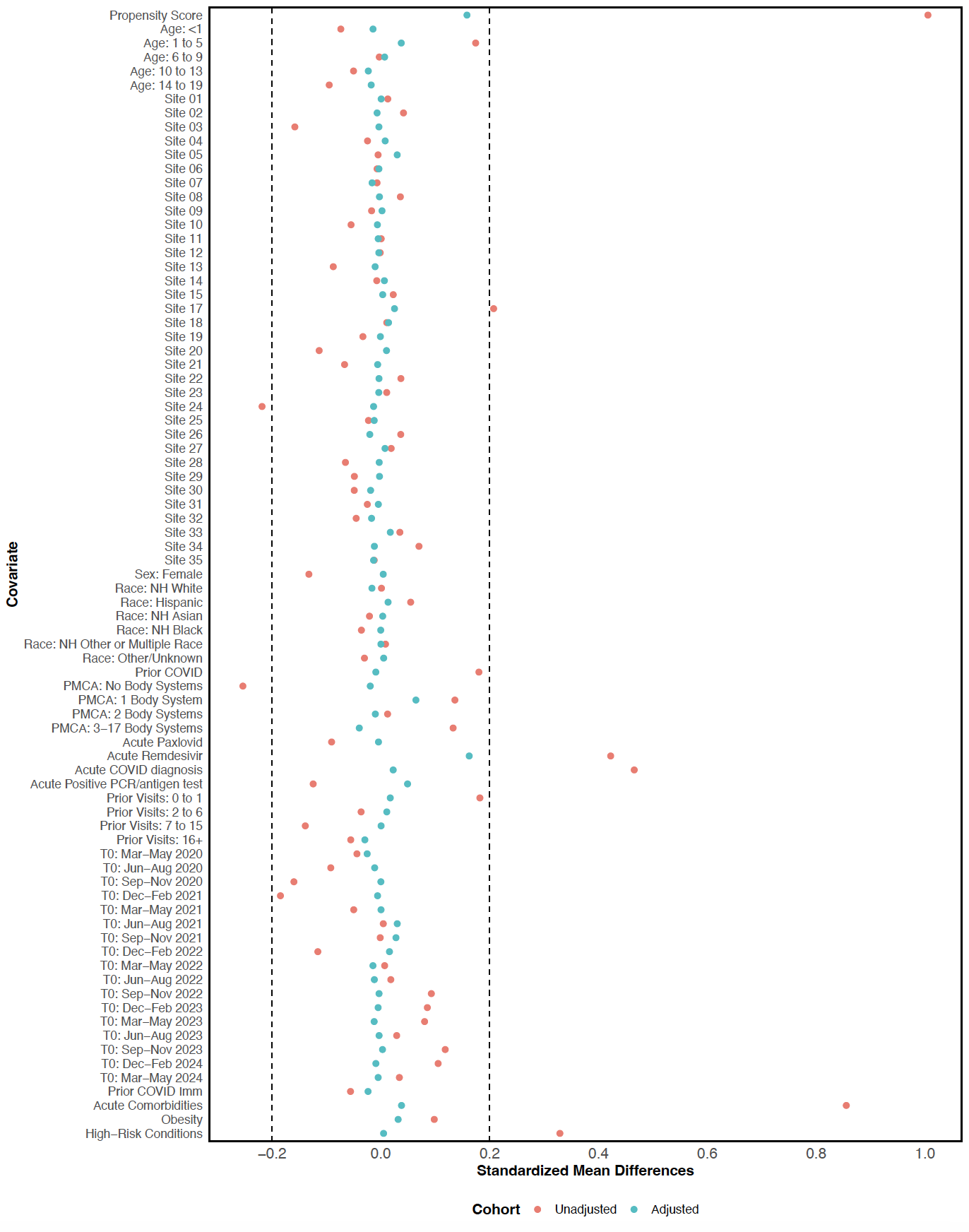


**S2 Fig. Density plot of post-truncated weights in treated and untreated groups among children and youth hospitalized with SARS-CoV-2 infection.**


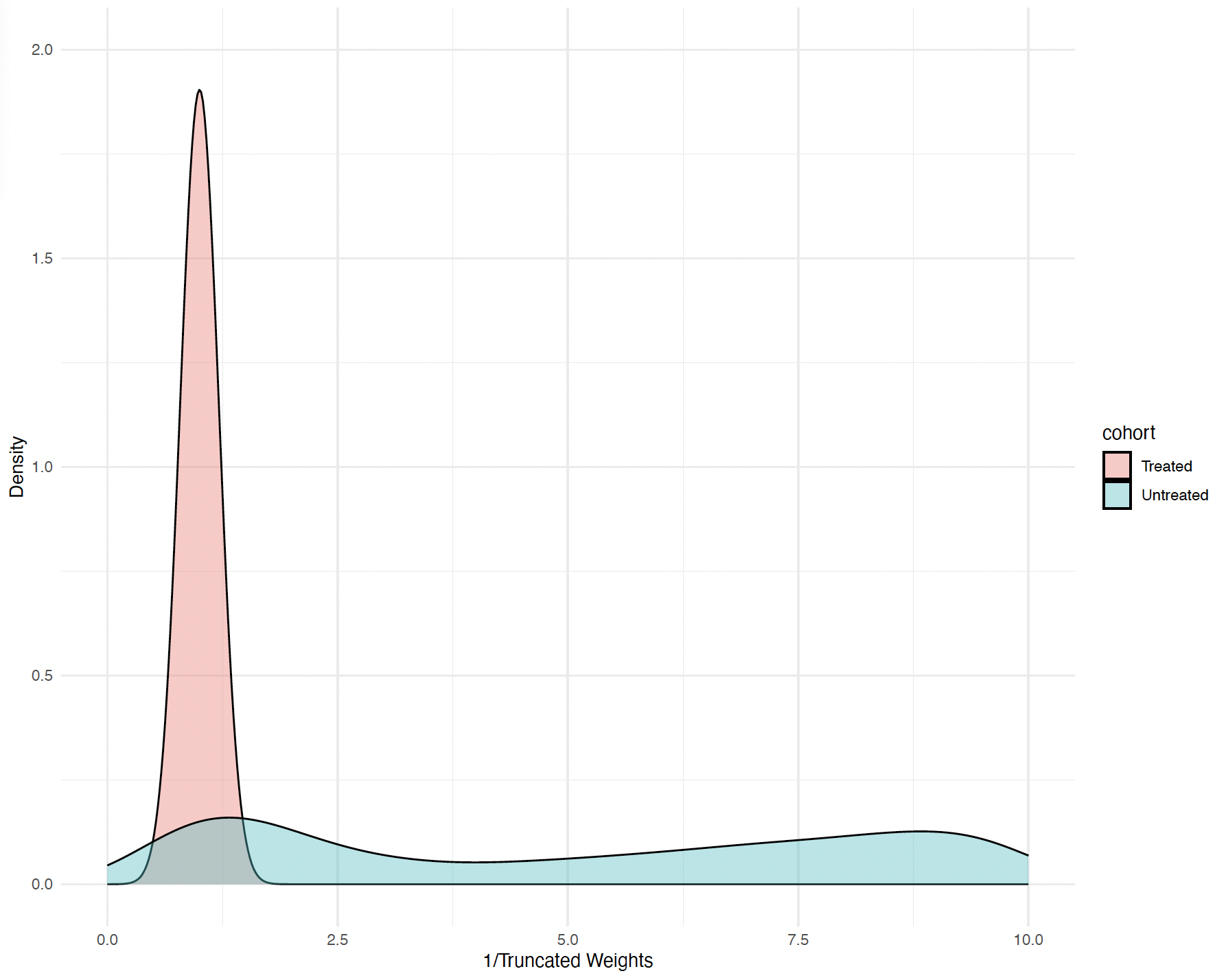


**S3 Fig. Love plot displaying standardized mean differences before and after applying inverse probability of treatment weighting (IPTW) in children and youth outpatients with SARS-CoV-2 infection.**


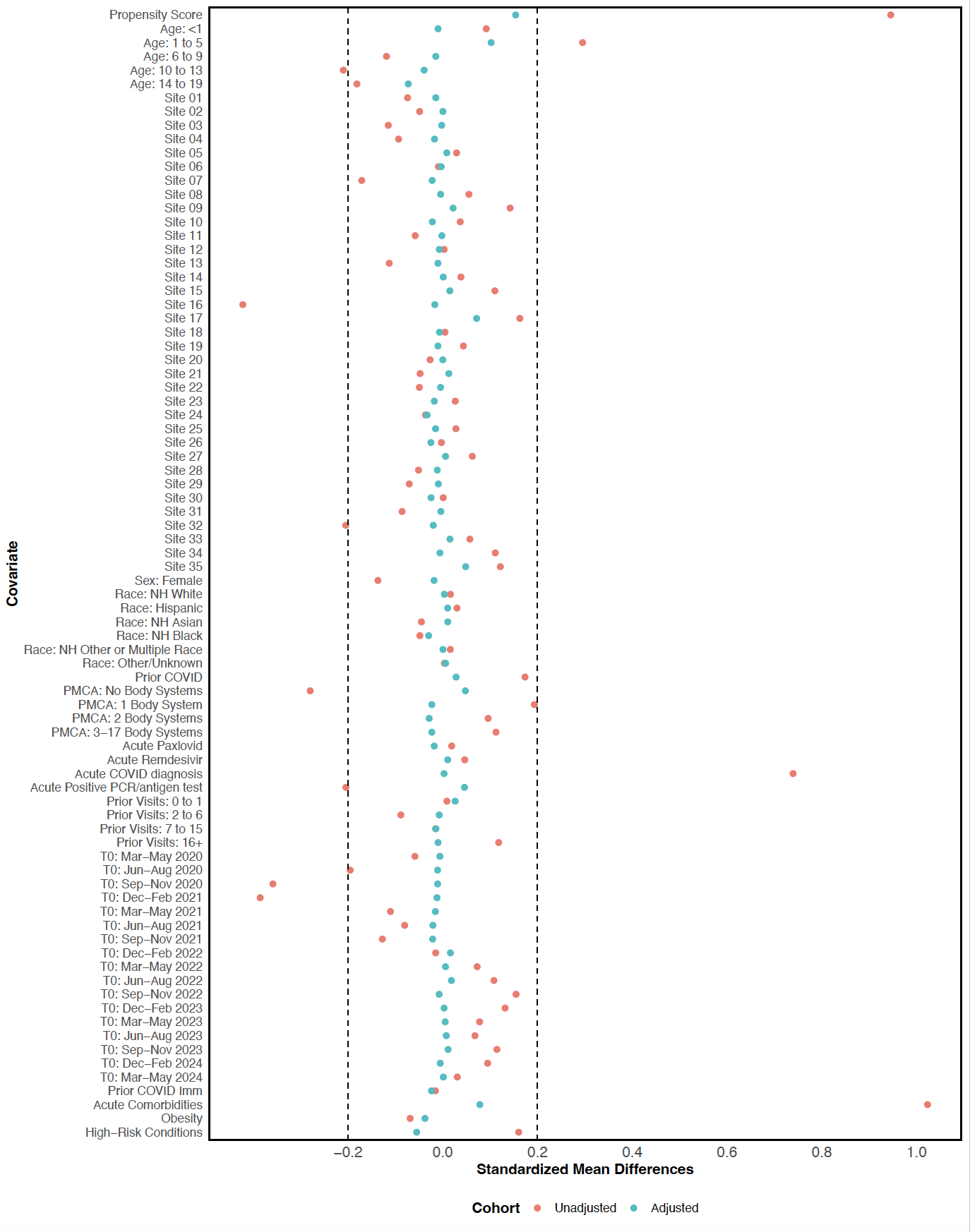


**S4 Fig. Density plot of post-truncated weights in treated and untreated groups among children and youth outpatients with SARS-CoV-2 infection.**


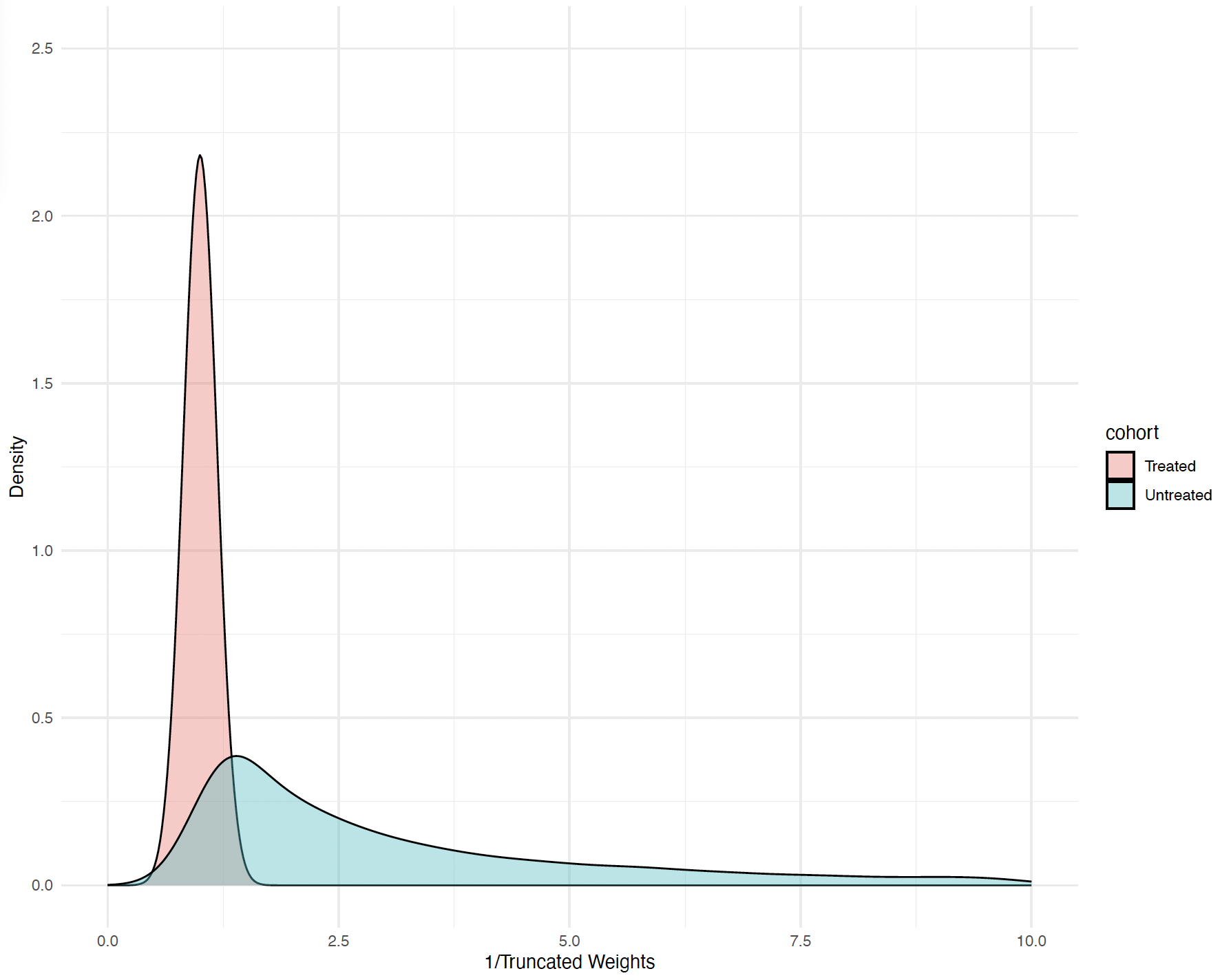


**S5 Fig. Cumulative incidence curves comparing the weighted cumulative incidence of PASC between the treated and untreated groups among children and youth hospitalized with SARS-CoV-2 infection.**


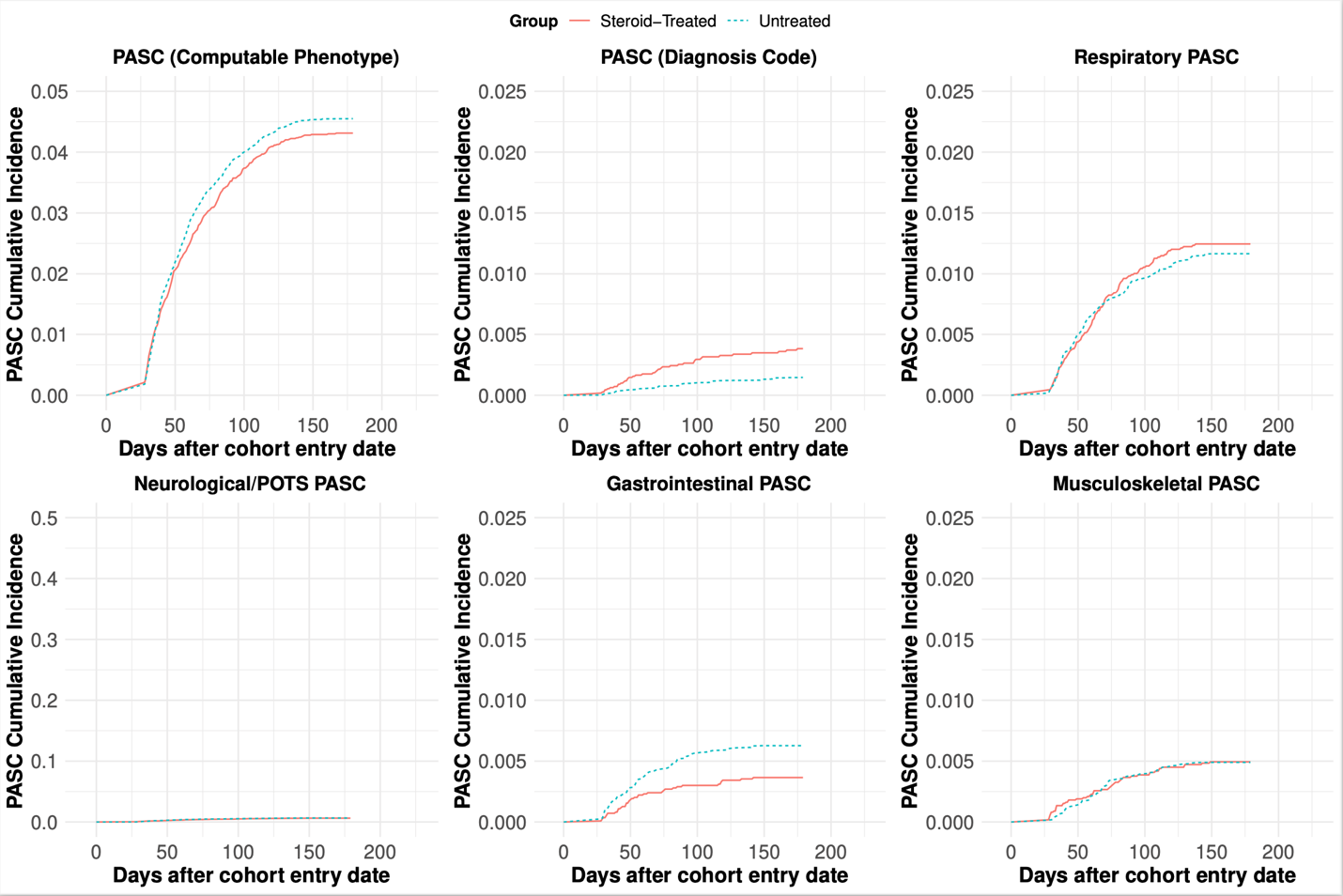


**S6 Fig. Cumulative incidence curves comparing the weighted cumulative incidence of PASC between the treated and untreated groups among children and youth outpatients with SARS-CoV-2 infection.**


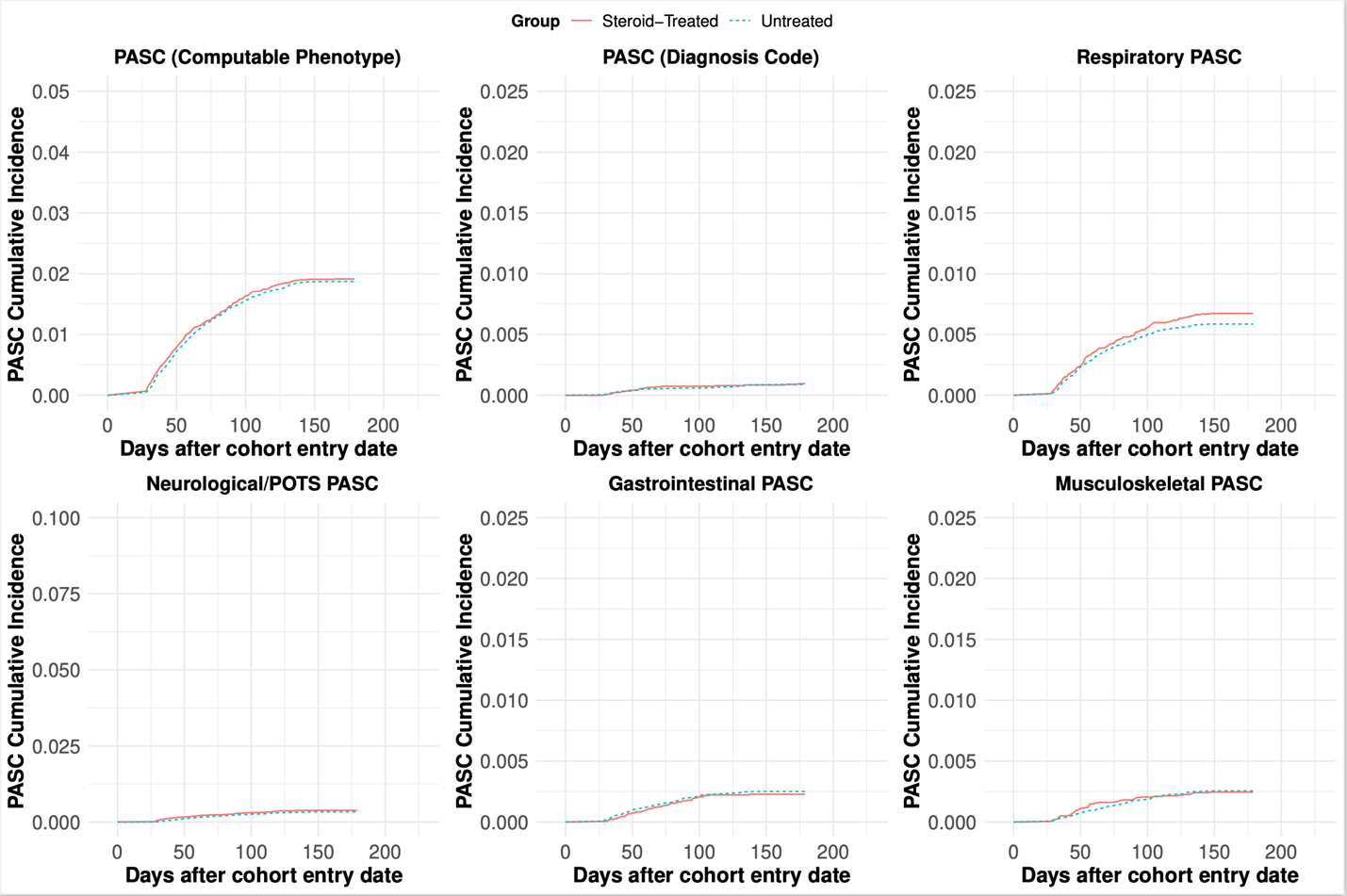


**S5 Table. Hazard Ratios from Cox proportional hazard models comparing risk of gastrointestinal PASC between the treated and untreated groups among children and youth with SARS-CoV-2 infection stratified by underlying gastrointestinal condition**

| **Stratification Group** | **Stratification Type** | **Treated N (weighted)** | **Not treated**  **N (weighted)** | **Hazard Rate (95% Confidence Interval)** | **P- Value** |
| --- | --- | --- | --- | --- | --- |
| **Underlying Gastrointestinal Condition** | Hospitalized during COVID, underlying gastrointestinal condition | 1,158 | 1,154 | 0.86 (0.40-1.85) | 0.71 |
|  | Hospitalized during COVID, no underlying gastrointestinal condition | 10,092 | 10,725 | 0.54 (0.35-0.83) | <0.01 |
|  | Outpatient during COVID, underlying gastrointestinal condition | 417 | 413 | 0.61 (0.13-2.83) | 0.53 |
|  | Outpatient during COVID, no underlying gastrointestinal condition | 21,668 | 21,484 | 0.93 (0.66-1.30) | 0.67 |

**S6 Table. Hazard Ratios from Cox proportional hazard models comparing risk of the PASC computable phenotype between the steroid-treated and untreated groups (hospitalized and outpatient) among children and youth with SARS-CoV-2 infection stratified by underlying gastrointestinal condition, age, COVID era, utilization within the year prior to the COVID date, excluding patients with steroid use within 180 days prior to COVID infection, and excluding patients with acute comorbidities.**

| **Stratification Group** | **Stratification Type** | **Treated N (weighted)** | **Not treated N (weighted)** | **Hazard Rate (95% Confidence Interval)** | **P- Value** |
| --- | --- | --- | --- | --- | --- |
| Age Group at T0 | Hospitalized during COVID, aged <5 years | 6,229 | 6,510 | 0.85 (0.71-1.02) | 0.08 |
|  | Hospitalized during COVID, aged 5 to 12 | 2,606 | 2,723 | 0.83 (0.65-1.07) | 0.15 |
|  | Hospitalized during COVID, aged 13+ | 2,415 | 2,366 | 1.03 (0.83-1.28) | 0.78 |
|  | Outpatient during COVID, aged <5 | 11,456 | 11,311 | 0.89 (0.73-1.07) | 0.22 |
|  | Outpatient during COVID, aged 5 to 12 | 5,756 | 5,793 | 0.98 (0.78-1.23) | 0.86 |
|  | Outpatient during COVID, aged 13+ | 4,873 | 4,904 | 1.29 (1.08-1.55) | 0.01 |
| COVID era | Hospitalized during COVID, pre-Omicron | 3,023 | 3,157 | 1.12 (0.90-1.40) | 0.32 |
|  | Hospitalized during COVID, in-Omicron | 8,227 | 8,515 | 0.89 (0.77-1.03) | 0.11 |
|  | Outpatient during COVID, pre-Omicron | 4,254 | 4,333 | 1.25 (0.99-1.60) | 0.07 |
|  | Outpatient during COVID, in-Omicron | 17,831 | 17,554 | 0.98 (0.86-1.12) | 0.79 |
| Utilization within the year prior to COVID infection | Hospitalized during COVID, low utilization (0 to 1 visits) | 4,307 | 4,590 | 1.20 (0.93-1.55) | 0.16 |
|  | Hospitalized during COVID, medium utilization (2 to 6 visits) | 2,329 | 2,514 | 1.07 (0.80-1.44) | 0.66 |
|  | Hospitalized during COVID, high utilization (≥7 visits) | 4,614 | 4,972 | 0.81 (0.70-0.95) | 0.01 |
|  | Outpatient during COVID, low utilization (0 to 1 visits) | 8,296 | 8,239 | 1.01 (0.76-1.34) | 0.93 |
|  | Outpatient during COVID, medium utilization (2 to 6 visits) | 6,367 | 6,277 | 1.04 (0.83-1.30) | 0.75 |
|  | Outpatient during COVID, high utilization (≥7 visits) | 7,422 | 7,454 | 1.08 (0.93-1.25) | 0.34 |
| Excluding steroid use within 180 days prior to COVID | Hospitalized during COVID, no steroid use within 180 days prior | 6,183 | 6,156 | 0.85 (0.73-1.00) | 0.06 |
|  | Outpatient during COVID, no steroid use within 180 days prior | 17,583 | 17,367 | 0.89 (0.77-1.02) | 0.08 |
| Excluding patients with acute comorbidities (asthma, bronchiolitis, croup, cystic fibrosis exacerbation) | Hospitalized during COVID, no acute comorbidities | 5,356 | 5,303 | 1.09 (0.94-1.25) | 0.25 |
|  | Outpatient during COVID, no acute comorbidities | 10,166 | 10,177 | 1.23 (1.08-1.42) | <0.01 |
| Including PEDSnet COVID-19 severity as variable in weighting | Hospitalized during COVID, including PEDSnet severity in weighting | 11,250 | 11,363 | 0.92 (0.81-1.04) | 0.20 |
|  | Outpatient during COVID, not including PEDSnet severity in weighting | 22,085 | 22,263 | 0.99 (0.88-1.11) | 0.82 |
| Underlying Gastrointestinal Condition | Hospitalized during COVID, underlying gastrointestinal condition | 1,158 | 1,155 | 0.87 (0.66-1.15) | 0.34 |
|  | Hospitalized during COVID, no underlying gastrointestinal condition | 10,092 | 10,725 | 0.96 (0.84-1.09) | 0.52 |
|  | Outpatient during COVID, underlying gastrointestinal condition | 417 | 413 | 0.92 (0.49-1.74) | 0.81 |
|  | Outpatient during COVID, no underlying gastrointestinal condition | 21,668 | 21,484 | 1.03 (0.92-1.16) | 0.58 |

a - As defined by PMCA gastrointestinal body system

**S7 Fig. Cox proportional hazard models comparing the steroid-treated group versus the untreated group in the development of PASC in the 1-6 months following acute infection within the hospitalized cohort, removing steroid use during follow-up and SARS-CoV-2 immunization during follow-up as censoring reasons.**

**
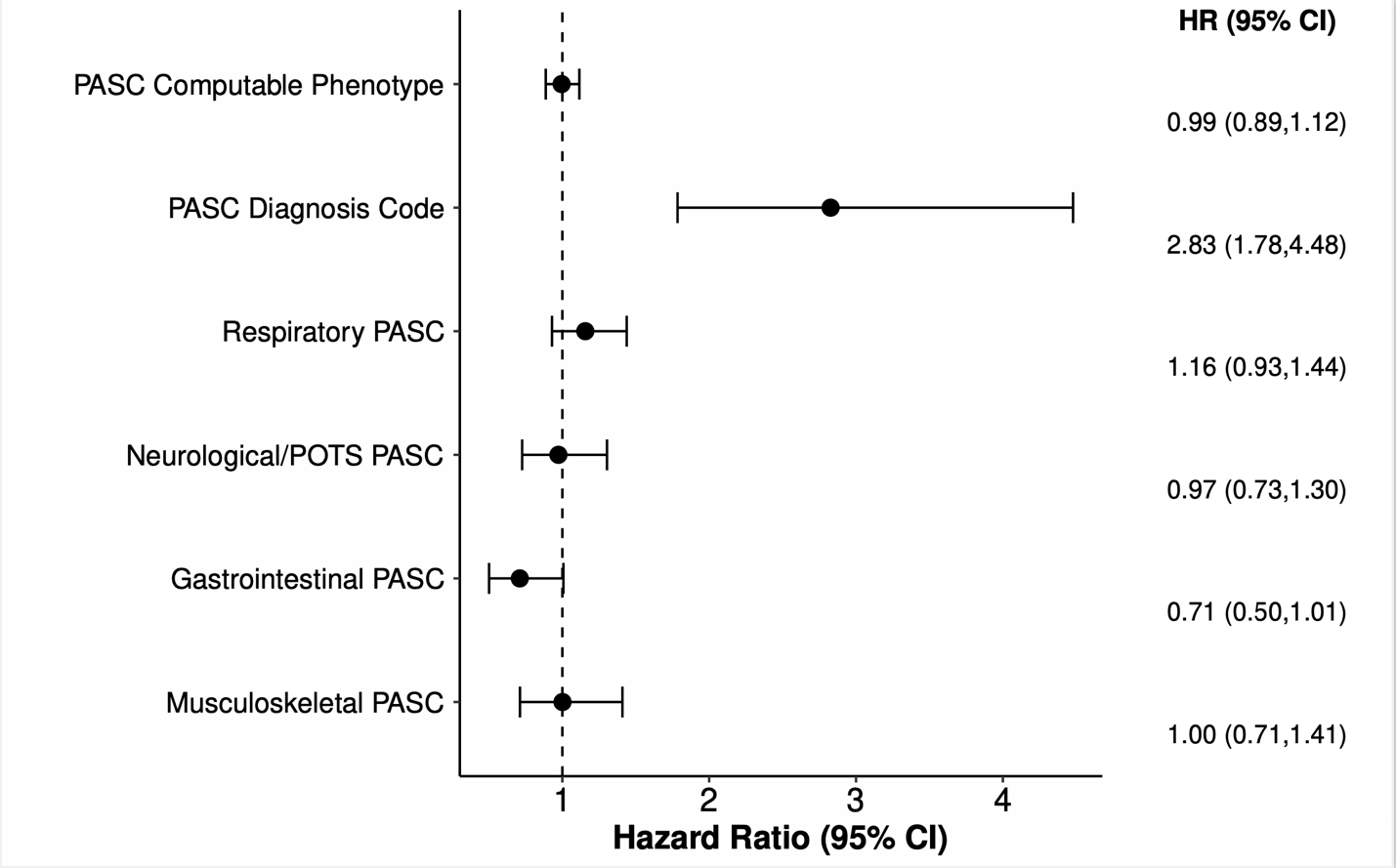
**

*The PASC diagnosis code refers to the U09.9 ICD-10 code.

**S8 Fig. Cox proportional hazard models comparing the steroid-treated group versus the untreated group in the development of PASC in the 1-6 months following acute infection within the outpatient cohort, removing steroid use during follow-up and SARS-CoV-2 immunization during follow-up as censoring reasons.**


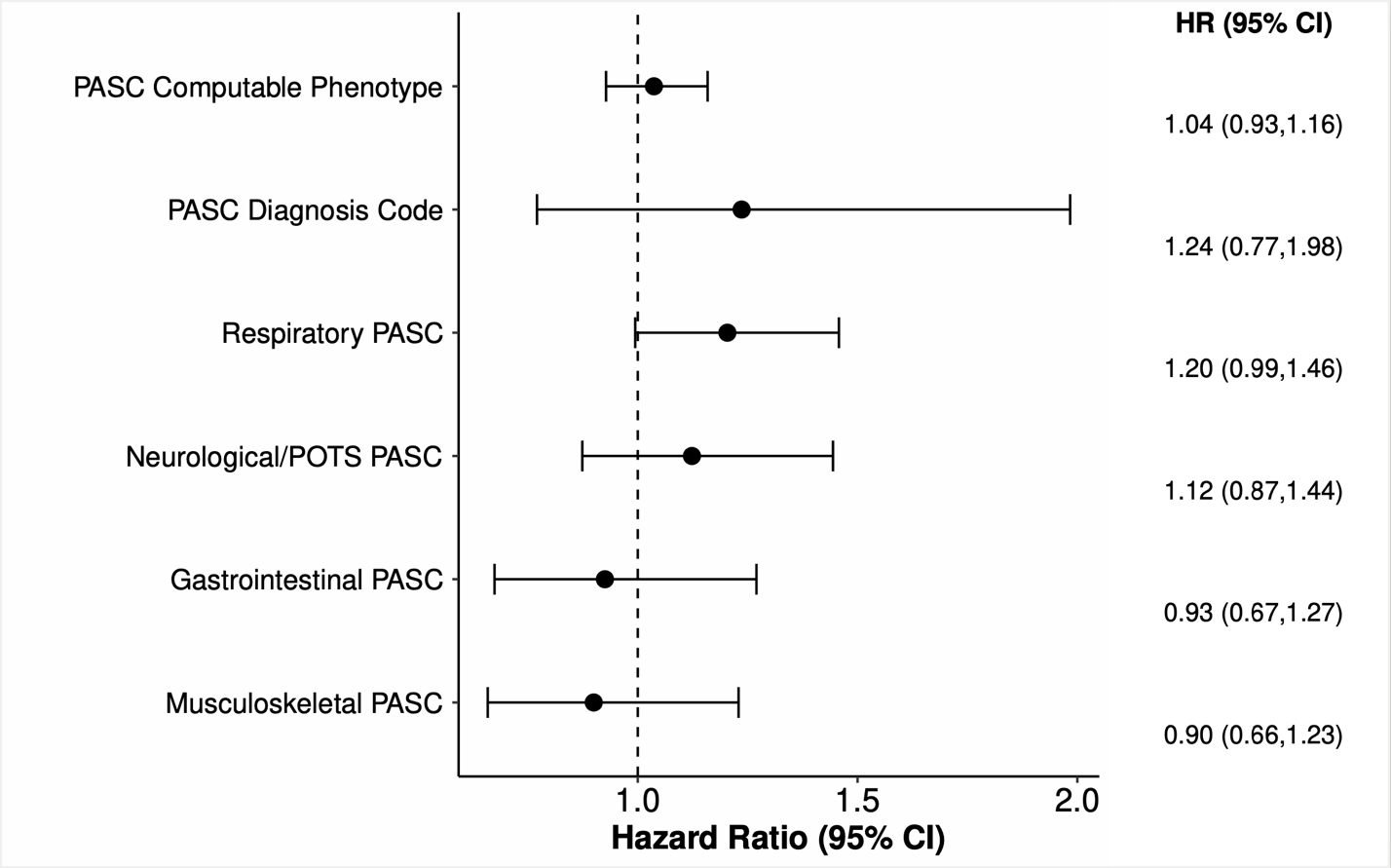


*The PASC diagnosis code refers to the U09.9 ICD-10 code.

**S9 Fig. Cox proportional hazard models comparing the steroid-treated group versus the untreated group in the development of PASC in the 1-6 months following acute infection within the hospitalized cohort, limiting to patients with dexamethasone use.**


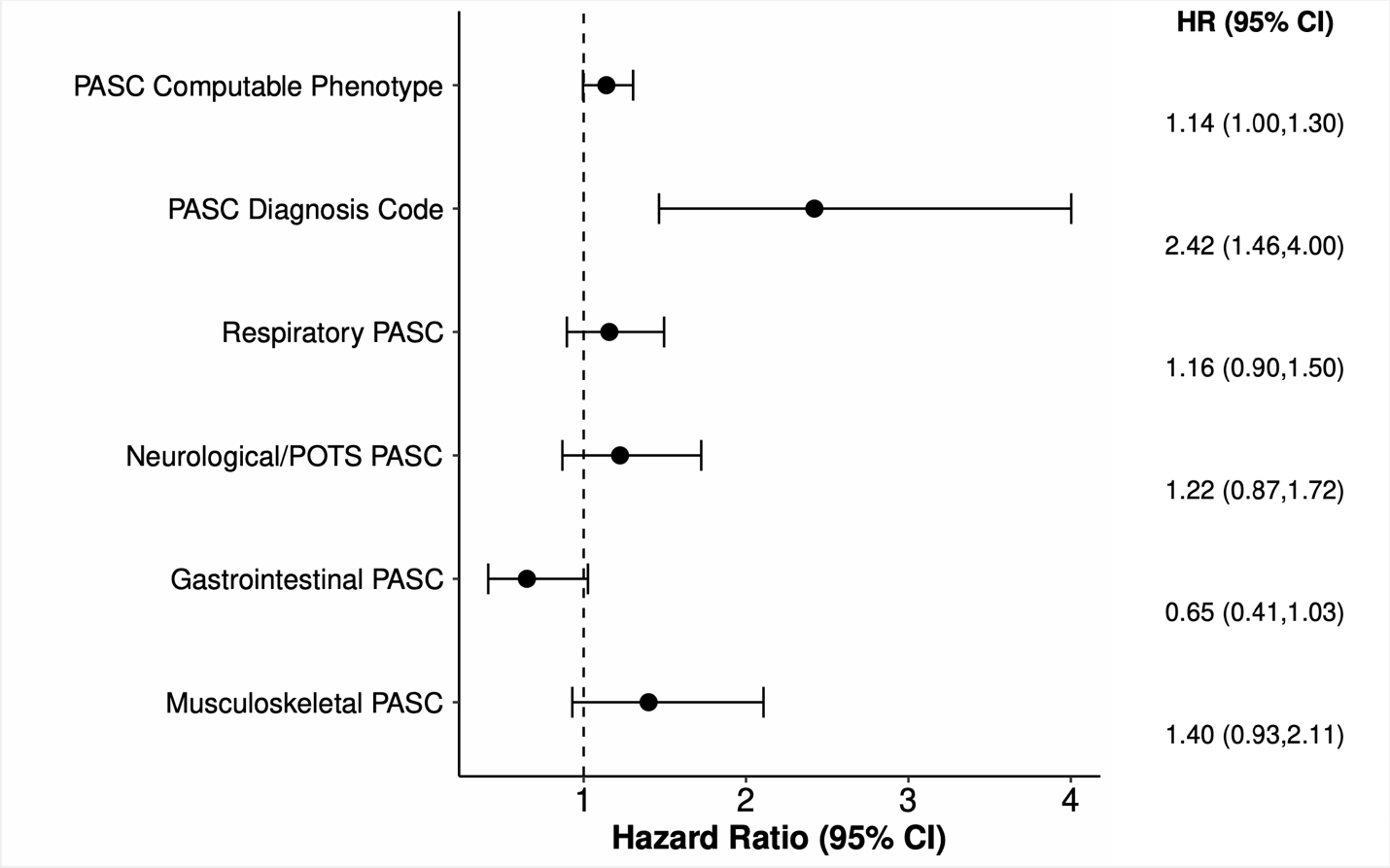


*The PASC diagnosis code refers to the U09.9 ICD-10 code.

**S10 Fig. Cox proportional hazard models comparing the steroid-treated group versus the untreated group in the development of PASC in the 1-6 months following acute infection within the outpatient cohort, limiting to patients with dexamethasone use.**


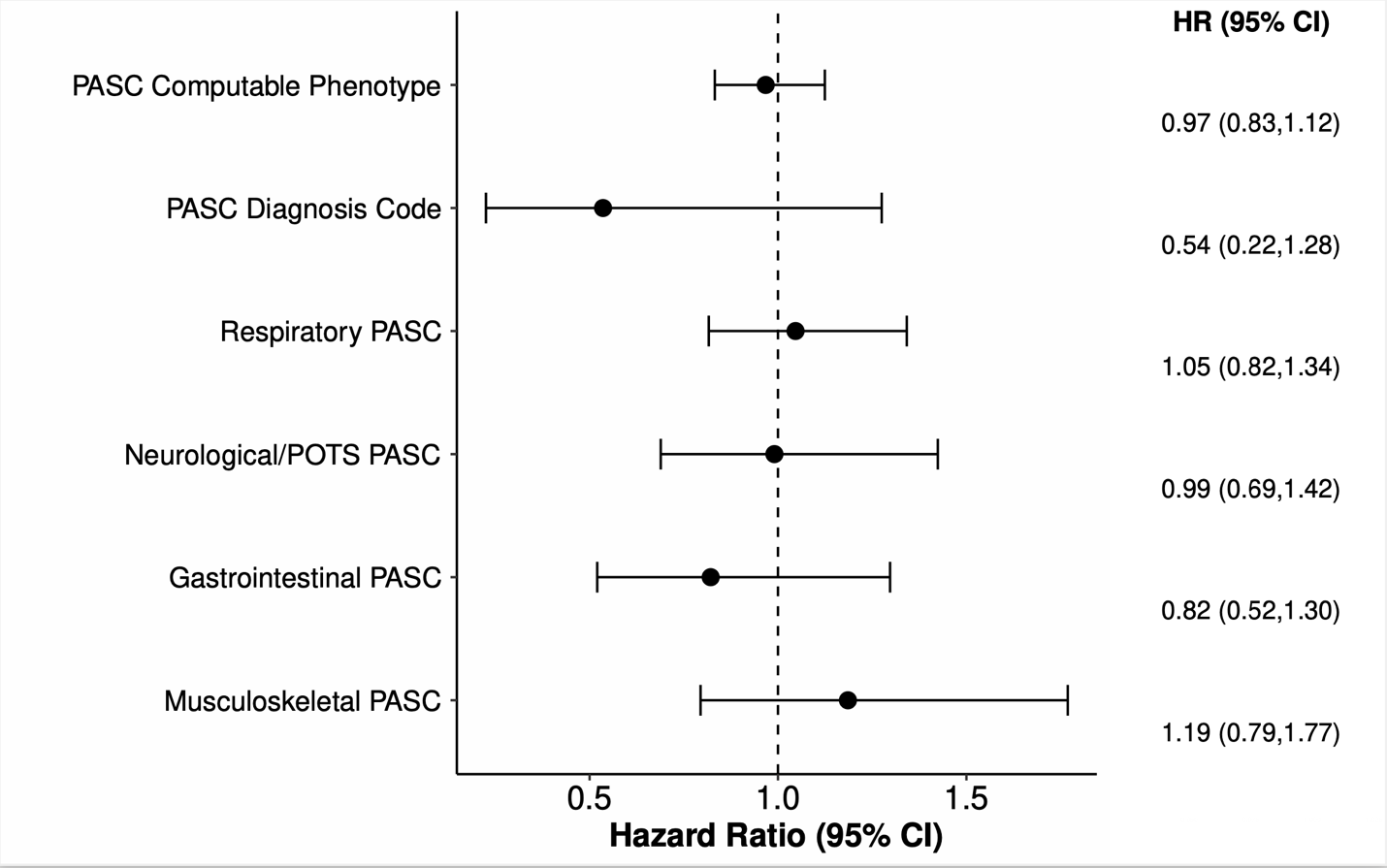


*The PASC diagnosis code refers to the U09.9 ICD-10 code.
