# Supplementary material for: Evaluation of steroids for acute COVID in the prevention of long COVID in children: An EHR and pediatric cohort study from the RECOVER Initiative
Source: PLoS One. 2026 Jun 18;21(6):e0350888. doi: 10.1371/journal.pone.0350888 (PMC13278399; doi:10.1371/journal.pone.0350888)
Supplement: Attachment — Submitted filename: Response to Reviewers.docx [file pone.0350888.s004.docx]

Resubmission of Manuscript PONE-D-25-56402: **“Evaluation of Steroids for Acute COVID in the prevention of Long COVID in Children: An EHR and Pediatric cohort study from the RECOVER Initiative.”**

**April 24, 2026**

**Dear Dr.** Bataduwaarachchi,

We thank you and the reviewers for the time and effort invested in evaluating our manuscript, *“Evaluation of Steroids for Acute COVID in the prevention of Long COVID in Children: An EHR and Pediatric cohort study from the RECOVER Initiative*” (Manuscript ID: PONE-D-25-56402). We appreciate the thoughtful and constructive comments, which have helped us to improve the clarity and rigor of the work.

We have carefully revised the manuscript and believe that we have fully addressed all concerns raised by the reviewers. Changes made to the manuscript are described in detail in the point-by-point response below. For clarity, reviewer comments are reproduced in regular text, followed by our responses in **bold.** All changes in the manuscript have been marked within the revised document [or tracked/highlighted, if applicable].

We believe that these revisions have strengthened the manuscript and clarified the study’s methodology, results, and interpretation. We hope that the revised version is now suitable for publication in PLOS ONE, and we appreciate your consideration of our resubmitted manuscript.

Please do not hesitate to contact us if additional information or clarification is required.

Sincerely,

**Miranda Higginbotham, MHA,** on behalf of all authors
Applied Clinical Research Center, Children’s Hospital of Philadelphia
higginbotm@chop.edu | 214-326-9432

**Response to Journal Requirement Comments**

1. Please ensure that your manuscript meets PLOS ONE's style requirements, including those for file naming. The PLOS ONE style templates can be found at <https://journals.plos.org/plosone/s/file?id=wjVg/PLOSOne_formatting_sample_main_body.pdf> and <https://journals.plos.org/plosone/s/file?id=ba62/PLOSOne_formatting_sample_title_authors_affiliations.pdf>
   - **Thank you for the information; we have updated the manuscript to meet PLOS ONE’s style and file naming requirements.**
2. When completing the data availability statement of the submission form, you indicated that you will make your data available on acceptance. We strongly recommend all authors decide on a data sharing plan before acceptance, as the process can be lengthy and hold up publication timelines. Please note that, though access restrictions are acceptable now, your entire data will need to be made freely accessible if your manuscript is accepted for publication. This policy applies to all data except where public deposition would breach compliance with the protocol approved by your research ethics board. If you are unable to adhere to our open data policy, please kindly revise your statement to explain your reasoning and we will seek the editor's input on an exemption. Please be assured that, once you have provided your new statement, the assessment of your exemption will not hold up the peer review process.
   - **We have updated our data availability statement to reflect the journal requirements and to align with wording used in recent PLOS ONE RECOVER publications. It also reflects NIH Data Management and Sharing (DMS) expectations and is internally consistent with standard IRB/ethics language used for RECOVER EHR analyses.**
   - **Updated Statement: *The results reported here are based on detailed individual-level patient data compiled as part of the RECOVER program. Due to the high risk of reidentification based on the number of unique patterns in the data, patient privacy regulations, including the Privacy Rule of the Health Insurance Portability and Accountability Act, as interpreted by the Privacy Offices of participating health systems, prohibit us from releasing the data publicly. The data are maintained in a secure enclave, with access managed by the program coordinating center to remain compliant with regulatory and program requirements for accountable data access. Access to deidentified data may be granted to qualified researchers who meet the criteria for access to confidential data through the RECOVER EHR Pediatric Coordinating Center (***[***recover@chop.edu***](mailto:recover@chop.edu)***), subject to review and approval and the execution of any required data use agreements. The corresponding author may be contacted to facilitate data access requests. All logic and analytic code used to define the study cohort and conduct the analyses are publicly available at:*** [***https://github.com/RECOVER-Coordinating-Center/steroids_long_covid***](https://github.com/RECOVER-Coordinating-Center/steroids_long_covid)
3. One of the noted authors is a group or consortium RECOVER Consortium. In addition to naming the author group, please list the individual authors and affiliations within this group in the acknowledgments section of your manuscript. Please also indicate clearly a lead author for this group along with a contact email address
   - **We have reached out the journal help desk about this, and it was determined this issue was erroneously flagged in the feedback we received. We were given the okay to include the list of consortium authors in its own document rather than the Acknowledgements. We were advised to include it as a 'Supporting Information' file type rather than 'Other. Communication information: Kit Stokes, Associate Peer Review Operations Specialist [plosone@plos.org.](mailto:plosone@plos.org) (Case Number: 09584510).  [Page 7, Line 186 (S1 Table)]**
4. Please include captions for your Supporting Information files at the end of your manuscript, and update any in-text citations to match accordingly. Please see our Supporting Information guidelines for more information: <http://journals.plos.org/plosone/s/supporting-information>.
   - **Supporting information caption formatting has been updated [Pages 30-32, Lines 543-593]**
5. If the reviewer comments include a recommendation to cite specific previously published works, please review and evaluate these publications to determine whether they are relevant and should be cited. There is no requirement to cite these works unless the editor has indicated otherwise
   - **We did not see any recommendations to cite specific previously published works**

**Response to Editor Comments**

1. How exactly was time zero defined for the emulated trial(s), and how did you prevent immortal time bias given the 12-day exposure ascertainment window and the 1–6-month outcome period
   - **Time zero (T0) was identified as the date of treatment initiation in patients who received steroids. The distribution of the difference in days between COVID infection date and T0 was identified in treated patients, and we sampled from this distribution to generate corresponding T0 dates in the untreated group. This information has been included in the “Cohorts” section of the results.[Pages 8-9, Lines 193-196]**
   - **We considered the 12-day exposure ascertainment window to be sufficiently narrow to reduce the likelihood of immortal time bias; we have included this note in the methods. [Page 9, Lines 201-202]**
2. Did you use artificial censoring for protocol deviations (e.g., untreated at assignment who later received steroids within the window), and were inverse-probability-of-artificial-censoring weights used?
   - **We used an intention-to-treat approach to identify treated and untreated patients. We identified patients who had steroids during acute COVID (+0 to +12 days, with a duration of 1 to 10 days). Protocol deviations after that date were not considered (a patient in the “untreated” category could not later enter the cohort as a “treated” patient). Additional details are included in S2 Table.[S2 Table (Steps 1, 2, 3, and 6)]**
   - **During the follow-up period, patients were right-censored at time of death, SARS-CoV-2 vaccination, remdesivir or nirmatrelvir/ritonavir use, or steroid use. In a sensitivity analysis, we removed steroid use or SARS-CoV-2 vaccination during the follow-up period as artificial censoring reasons, to address concerns about potential informative censoring (e.g., patients who receive steroids during follow-up may be more likely to develop PASC but may be censored prior to the development of PASC). Results were similar, though the decreased risk of GI PASC for treated patients in the hospitalized cohort was no longer significant. Considering the similarity of the results with different censoring assumptions, especially regarding our main outcomes of interest (the PASC computable phenotype and diagnosis code), we find our censoring approach to be reasonable. Inverse-probability-of-artificial-censoring weights were not applied. [Page 22, Lines 350-353]**
3. What covariates entered the propensity model, how were missing data handled, and what were the pre/post-weight standardised mean differences for key variables (severity proxies, site, calendar time, vaccination)?
   - **Covariates included in the propensity models are described in the “Covariates and Stratification” section of the methods. [Page 10, Lines 216-231]**
   - **Table S2 and S3 include information about the exclusion of patients with missing or improbable data from the cohort (e.g., patients with negative age at their COVID cohort entry date). For the PMCA, patients who did not have any indication of body systems in the 3 years prior to T0 were included in the “No body systems” category. For the sex variable, a footnote has been added to describe the “Other/unknown/ambiguous” category. For the race/ethnicity variable, a footnote has been added to describe the “Other/Unknown” category. [S2 Table (Step 4); S3 Table (Steps 2, 4 and 6)]**
   - **Standardized mean differences after weighting are included in Table 1 and Table 2 and are visualized before and after weighting in Figures S1 and S3. Density plots have also been included in Figures S2 and S4 [Pages 12-13, Lines 281-283]**
4. Please provide weight diagnostics (stabilisation, truncation thresholds, distributional plots, and effective sample size) and positivity assessments for both inpatient and outpatient cohorts.
   - **The weighted samples, including effective sample sizes and post-weighting standardized mean differences, are included in Table 1 and Table 2; love plots and density plots are displayed in Figures S1-S4. Weights were considered extreme if they were >99.5^th^ or <0.5^th^ percentile and were redefined using the values of weights at the 99.5^th^ or 0.5^th^ percentile, and this information is listed in the methods. [Page 11, Lines 238-244 and Pages 12-13, Lines 281-283]**
5. How were non-COVID indications for steroids (e.g., asthma exacerbations, autoimmune flares) identified and handled? Can you provide sensitivity analyses excluding these indications?
   - **During balancing, we included a variable to identify patients with diagnoses that could be indications for steroids during the acute period (asthma, croup, bronchiolitis, cystic fibrosis exacerbation). [Page 10, Line 222-223]**
   - **A sensitivity analysis excluding patients with acute comorbidities is included in Table S5; the analysis showed no difference in patients who were hospitalized during COVID, and a slightly increased risk of PASC in steroid-treated patients who were not hospitalized during COVID (HR: 1.23, 95% CI: [1.08-1.42], p < 0.01). However, results were considered exploratory and were not adjusted for multiple comparisons. [Page 22, Lines 343-346]**
6. What were the steroid routes, doses and durations across agents, and can you analyze dose-response or regimen-specific effects?
   - **Dose and duration were not reliably indicated in the EHR for our examination, and we have included a note about our inability to address dose or duration (including dose-response effects) in the limitations. [Page 25, Lines 415-417]**
   - **In a sensitivity analysis, we were able to examine the effects of dexamethasone specifically; however, due to a limited sample size, we lacked sufficient power to examine multiple types of treatment regimens. [Pages 22-23, Lines 354-358]**
   - **Only oral or injection routes of steroid exposure were included; we have added the percentage of exposures indicated as oral and injection in the demographic tables (Table 1 and Table 2).**
7. How was the pediatric PASC computable phenotype validated in your networks (PPV, sensitivity) and how did U09.9 adoption timing and site practices influence ascertainment? Please provide sensitivity analyses using alternative definitions and windows (e.g., 2–6 months).
   - **Additional information about the validation of the PASC rules-based computable phenotype is included in the referenced article by Botdorf et al. (2025): Identifying Pediatric Long COVID: Comparing an EHR Algorithm to Manual Review. The computable phenotype shows moderate agreement with clinician identification (PPV = 0.49, NPV = 0.75, sensitivity = 0.52, specificity = 0.84). In the discussion of limitations, we have additionally noted that the computable phenotype has decreased performance for youth with underlying comorbidities, which can lead to misclassification of long COVID. [Page 26, Lines 423-424 and Table 3]**
   - **The U09.9 code comprised only a small percentage of outcomes identified via the PASC computable phenotype, as indicated by the differences in incidence rates between the computable phenotype and diagnosis code definitions (Table 3). Most PASC outcomes were identified through symptom clusters. Differences in U09.9 adoption timing and site practices changed over the course of the pandemic; this issue has been included as a limitation in the discussion. [Page 26, Lines 424-427]**
8. Given multiple subphenotype tests, did you adjust for multiplicity? Does the GI subphenotype association remain after correction and in stratified/sensitivity analyses?
   - **We have added a note to describe the subphenotype analyses as secondary, exploratory outcomes; we also noted that the GI subphenotype association was not significant after Bonferroni correction. The additional sensitivity/stratified analyses were considered exploratory and were not corrected for multiple comparisons; we have noted this in the “Sensitivity Analyses” section of the methods. [Page 9, Line 213, Page 12, Lines 270-271, Page 21, Lines 318-320]**
9. Were subgroup analyses by age, variant era, vaccination status, and inpatient severity (ICU, ventilation) conducted, and do any suggest effect modification?
   - **We performed sensitivity analyses stratified by age group, COVID era, and accounting for SARS-CoV-2 severity as a variable in weighting; only about 10% of the cohorts were vaccinated, so stratification by vaccination status may be underpowered to detect effects. We reported on subgroup analyses in the “Sensitivity Analyses” results section. [Pages 11-12, Lines 255-271]**
   - **We observed an increased risk of the PASC computable phenotype in outpatients aged 13 and older who were treated with steroids; no differences in effects were observed when stratifying by COVID pre-Omicron or Omicron era. Our cohorts were underpowered to perform analyses stratified by inpatient severity, but we did not observe differences in effects when accounting for SARS-CoV-2 severity in weighting. Sensitivity analyses were considered exploratory and results were not corrected for multiple comparisons. [Pages 22-23, Lines 338-358]**
10. Can you provide absolute risks and risk differences for primary and secondary outcomes to complement hazard ratios and aid clinical interpretation?
    - **The weighted incidence rates are intended to complement the hazard ratios and include person-year denominators rather than patient count denominators. [Page 19, Lines 303-308]**

**Response to Reviewer 1**

1. Index date and time zero: Please specify, in a reproducible manner, how COVID date is selected when there are multiple candidate events and how T0 is assigned to exposed vs unexposed. Confirm that all baseline covariates are measured strictly pre-T0 to avoid time bias due to time origin and immortality
   - **A random date of COVID was selected for patients who had steroids during their (+0 to +12-day) acute COVID period around cohort entry date, and a random date of COVID was selected for untreated patients with no evidence of steroids around the cohort entry date. Time zero (T0) was identified as the date of treatment initiation in patients who received steroids. The distribution of the difference in days between COVID infection date and T0 was identified in treated patients, and we sampled from this distribution to generate corresponding T0 dates in the untreated group. The detailed cohort inclusion criteria in Table S2 include step-by-step information about the identification of COVID dates and T0 dates. All time-dependent covariates were measured on or prior to T0; we have added a note to clarify this point in the methods. [Pages 8-9, Lines 193-196, Page 9, Lines 202-204, S2 Table (Steps 4, 5 and 7), and Page 10, Line 231]**
2. Informative censoring: Censoring at vaccination/antivirals/subsequent steroids may be outcome-related, and conclusions appear sensitive to censoring choices. Please justify censoring assumptions and consider IPCW or a structured sensitivity analysis.
   - **Analyses were rerun removing steroid use or SARS-CoV-2 vaccination during the follow-up period from the list of censoring reasons, to address concerns about potential informative censoring (e.g., patients who receive steroids during follow-up may be more likely to develop PASC but may be censored prior to the development of PASC). Results were similar, though the decreased risk of GI PASC for treated patients in the hospitalized cohort was no longer significant. Considering the similarity of the results with different censoring assumptions, especially regarding our main outcomes of interest (the PASC computable phenotype and diagnosis code), we find our censoring approach to be reasonable. [Page 22, Lines 350-353]**
3. Severity/confounding by indication: Residual confounding is likely for pediatric steroid prescribing. Please strengthen severity control (e.g., include severity proxies in weighting, restrict/stratify by severity) and report balance on key severity measures
   - **We successfully balanced (|SMD| <0.2) on acute comorbidities in our main model to better account for severity of illness during COVID. To strengthen severity control, we also balanced on a PEDSnet variable for SARS-CoV-2 severity during sensitivity analyses; results are included in Table S5. No differences in risk of the PASC computable phenotype were observed when including the PEDSnet variable for SARS-CoV-2 severity in weighting (hospitalized HR: 0.92; [95% CI: 0.81-1.04]; outpatient HR: 0.99; [95% CI: 0.88-1.11]). [Pages 12-13, Lines 281-283, and Page 22, Lines 346-349]**
4. Outcome validity / misclassification: Please provide validity evidence or proxy checks by strata and, if possible, report post-acute healthcare utilization to quantify detection bias.
   - **For context, we added variables indicating average counts of visits occurring 28-179 days after T0 and 180-365 days after T0 to Tables 1-2. In both hospitalized and outpatient cohorts, post-acute utilization appeared similar up to a year after T0. [Table 1 and 2]**
5. Multiple test corrections: With multiple secondary outcomes/sensitivity analyses (no correction), the isolated hospitalized GI signal should be framed as exploratory. Consider FDR control or a pre-specified outcome hierarchy.
   - **We updated language in the manuscript to describe the secondary PASC subphenotype outcomes as exploratory and described the finding of reduced risk of gastrointestinal PASC as non-significant following Bonferroni correction. [Page 9, Line 213, Page 21, Lines 318-320]**
6. Report IPTW diagnostics: weight distribution, % trimmed, effective sample size
   - **The weighted samples, including effective sample sizes and post-weighting standardized mean differences, are included in Table 1 and Table 2; love plots and density plots are displayed in Figures S1-S4. Weights were considered extreme if they were >99.5^th^ or <0.5^th^ percentile and were redefined using the values of weights at the 99.5^th^ or 0.5^th^ percentile, and this information is listed in the methods. [Page 11, Lines 238-244]**
7. Consider site heterogeneity: site-stratified models or frailty
   - **We balanced on site when performing IPTW. Given the small counts of patients per site and the fact that no site comprised greater than 15% of the cohort, we considered site-stratified models to be underpowered. [Page 10, Line 219]**
8. Clarify data/code access: with a concrete pathway and a versioned, archived code release
   - **We have added a publicly accessible link to the Github repository. [Page 9, Line 212]**

**Response to Reviewer 2**

1. Several methodological issues should be considered when interpreting the findings. First, confounding by indication remains possible. Children who received steroids were likely more severely ill or had stronger clinical indications for treatment, which may still influence the risk of long COVID despite statistical adjustment
   - **While we attempted to mitigate confounding by indication through the target trial emulation approach and weighting, we agree that confounding by indication remains possible. We have noted this issue in the limitations.[Page 25, Lines 417-419]**
2. Second, the classification of steroid exposure appears overly broad, as different corticosteroids (such as dexamethasone, prednisone, prednisolone, and methylprednisolone) vary in potency, route of administration, and clinical indications. Treating them as a single group may mask important differences.
   - **In consideration of the broad classification of steroid exposures, we included a sensitivity analysis in which only dexamethasone was evaluated as a steroid exposure. We have also noted the classification of steroid exposure using multiple steroid types as a study limitation. [Pages 22-23, Lines 354-358, Page 25, Lines 415-417]**
3. In addition, the analysis does not sufficiently address dose, duration, or route of steroid therapy, which limits the biological interpretation of the results.
   - **Dose and duration were not reliably indicated in the EHR for our examination, and we have included a note about our inability to address dose or duration in the limitations. Only oral or intravenous injection routes of steroid exposure were included; we have added the percentage of exposures indicated as oral and injection in the demographic tables (Table 1 and Table 2). [Page 25, Lines 415-417, Page 9, Lines 198-199]**
4. Another concern relates to the definition of long COVID, which relies on a computable EHR phenotype. This approach may lead to misclassification, particularly for mild or non-specific symptoms.
   - **Our approach using a computable phenotype, as described earlier, is a much more reliable capture than long COVID ICD 10 codes, but it is possible that milder long COVID symptoms have not been captured in our cohort, especially given the phenotype’s decreased performance in youth with underlying comorbidities. We have acknowledged this in the limitations. [Page 26, Lines 423-424]**
5. There is also a possibility of follow-up bias, as children who received steroids may have had more clinical visits and therefore more opportunities to receive PASC-related diagnoses
   - **For context, we added variables indicating average counts of visits occurring 28-179 days after T0 and 180-365 days after T0 to Tables 1-2. In both hospitalized and outpatient cohorts, post-acute utilization appeared similar up to a year after T0. [Table 1 and Table 2]**
6. Finally, the study spans multiple COVID-19 periods, during which variants, vaccination status, and clinical practices changed substantially. These factors introduce additional heterogeneity. For these reasons, the conclusions should be presented with slightly more caution.
   - **We attempted to account for the T0 period during weighting; in sensitivity analyses, we also examined results stratified by pre-Omicron and in-Omicron era, though we did not observe differences. Nonetheless, there were substantial changes in variants, vaccination status, and clinical practices over the course of the study; we have included this as a study limitation. [Page 10, Line 219, Page 22, Lines 346-347, Page 26, Lines 424-427]**
